# Controlling the volatility of the written optical state in electrochromic DNA liquid crystals

**DOI:** 10.1038/ncomms11476

**Published:** 2016-05-09

**Authors:** Kai Liu, Justin Varghese, Jennifer Y. Gerasimov, Alexey O. Polyakov, Min Shuai, Juanjuan Su, Dong Chen, Wojciech Zajaczkowski, Alessio Marcozzi, Wojciech Pisula, Beatriz Noheda, Thomas T. M. Palstra, Noel A. Clark, Andreas Herrmann

**Affiliations:** 1 Zernike Institute for Advanced Materials, University of Groningen, Nijenborgh 4, 9747 AG Groningen, The Netherlands; 2 Department of Physics and Soft Materials Research Center, University of Colorado, Boulder, Colorado 80309, USA; 3 Institute of Process Equipment, College of Chemical and Biological Engineering, Zhejiang University, Hangzhou 310027, China; 4 Max Planck Institute for Polymer Research, Ackermannweg 10, 55128 Mainz, Germany; 5 Department of Molecular Physics, Faculty of Chemistry, Lodz University of Technology, Zeromskiego 116, 90-924 Lodz, Poland

## Abstract

Liquid crystals are widely used in displays for portable electronic information display. To broaden their scope for other applications like smart windows and tags, new material properties such as polarizer-free operation and tunable memory of a written state become important. Here, we describe an anhydrous nanoDNA–surfactant thermotropic liquid crystal system, which exhibits distinctive electrically controlled optical absorption, and temperature-dependent memory. In the liquid crystal isotropic phase, electric field-induced colouration and bleaching have a switching time of seconds. Upon transition to the smectic liquid crystal phase, optical memory of the written state is observed for many hours without applied voltage. The reorientation of the DNA–surfactant lamellar layers plays an important role in preventing colour decay. Thereby, the volatility of optoelectronic state can be controlled simply by changing the phase of the material. This research may pave the way for developing a new generation of DNA-based, phase-modulated, photoelectronic devices.

## Abstract (web summary)

Electrochromism, the dependence of light absorption upon electronic control, finds a wide range of applications in smart materials. Here, Liu *et al*. show an electrochromic DNA–surfactant liquid crystal system that exhibits electrically tunable optical absorption and thermally tunable memory.

## Graphical abstract

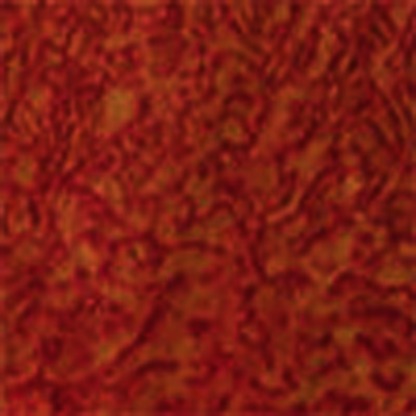

Electrochromism, or the electrical control of a material's absorption of light^1^,^2^,^3^, has led to the development of many technological applications that span dynamic tinting windows and mirrors, colour changing displays, smart cards and e-paper^4^. Several distinct classes of materials have been extensively investigated for their usefulness in electrochromic applications, including organic systems (for example, bipyridylium salts)^5^,^6^,^7^,^8^,^9^, electroactive conducting polymers (for example, polyaniline, polythiophenes)^1^,^10^,^11^,^12^,^13^, metal-organic systems (for example, Prussian blue, Fe-terpyridines coordination polymer, Zn-pyrazolate metal–organic frameworks)^14^,^15^,^16^,^17^ as well as inorganic systems based on transition metal oxides (for example, WO_3_)^18^,^19^,^20^,^21^. Despite these advances, there is a strong need for more accessible and easily processable materials with a greater degree of tunability of electrochromic behaviour. Challenges to be achieved include control over the duration of an electronic state in the absence of electrical input or in response to temperature changes. Therefore, the development of new electrochromic materials is an important goal.

Liquid crystals (LCs) are phases of soft condensed matter in which varying degrees of partial orientational and positional ordering result in an enormous variety of functional properties^22^,^23^. The flexibility inherent in LC physical organization, along with that of organic molecular electronic structures for redox-active functionality, combine to form an attractive basis for pursuing electrochromism^24^,^25^,^26^. Furthermore, this flexibility in LC ordering may lead to novel electrochromic behaviours different from those of disordered electrochromic materials.

Thus far, the unique electrical characteristics of DNA have prompted the utilization of DNA in the fields of materials and nanotechnology^27^,^28^,^29^. However, little effort has been invested in studying the electrochromic properties of DNA, which is appealing as an electrochromic material due to its ubiquity and uniform distribution of redox-active sites (nucleobases) along its polymer backbone^30^,^31^,^32^. Recently, we have prepared a series of thermotropic LCs based on DNA–surfactant complexes that are produced by a simple preparation protocol^33^,^34^. Phase transition temperatures from the isotropic liquid phase to the LC phase to the crystalline phase can be modulated over a broad temperature range by controlling the average length of the cationic surfactant that is complexed to the negatively charged single-stranded nucleic acid molecule.

Here, we report the development of DNA-LC-based electrochromic devices that are readily switchable between the coloured and colourless states in the isotropic phase. The rate of colouration decay can be regulated by adjusting the phase of the DNA–surfactant complex, the length of the DNA oligomer and the identity of the complexing surfactant. A colour impression can thus be preserved for several hours in the absence of applied voltage if the temperature of the system is adjusted to accommodate the LC phase or the crystalline phase. Moreover, in response to temperature changes above the clearing temperature, immediate colour loss is observed. This phenomenon allows for controlling the volatility of stored information in a simple and practical manner and might allow the fabrication of smart tags with clock and thermometer functions.

## Results

### Preparation and characterization of DNA–surfactant LCs

**Figure 1:**
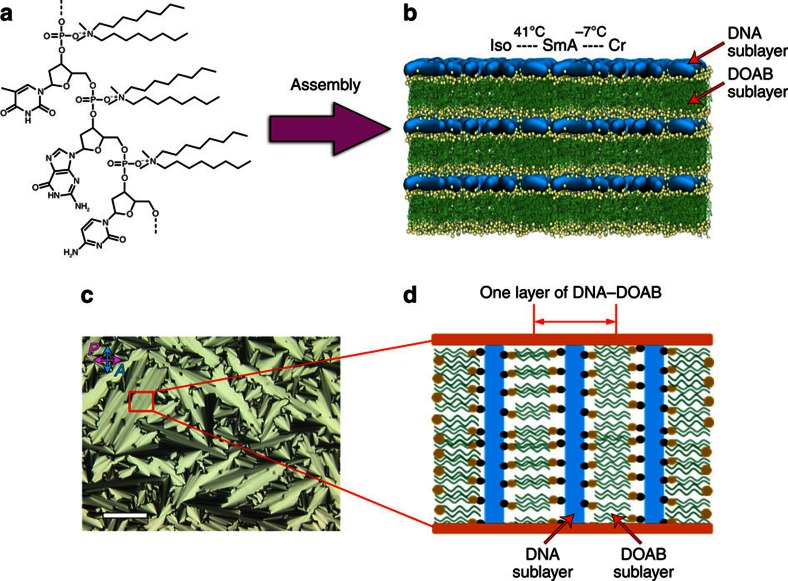
Schematic illustration of the DNA–surfactant complex formation. (**a**) Molecular structure of DNA–surfactant complex using dioctyldimethylammonium bromide (DOAB) as a representative cationic surfactant that electrostatically interacts with the oligonucleotide backbone. With increasing temperature, the DNA–surfactant complexes transition from crystalline state to the LC state and further to the isotropic liquid state. (**b**) The lamellar bilayer structure in the LC phase is made of one sublayer of single-stranded DNA and one sublayer of interdigitated surfactants, where the phosphate groups of DNA electrostatically interact with the cationic head groups of surfactants. (**c**) A polarized optical microscopy (POM) image of the DNA–surfactant LC phase in a LC cell (here taking 14mer DNA–DOAB as a representative complex, 25 °C, cell gap ∼6.8 μm) shows well-defined focal-conic textures of the smectic phase. Scale bar, 100 μm. (**d**) An illustration of the alignment of the DNA–surfactant LC in the cell shows that the DNA–surfactant smectic layers are vertical to the ITO-coated glass slide surface. In **d**, black ball represents phosphate anions of oligonucleotide and yellow ball represents ammonium cations of surfactant.

DNA–surfactant materials were prepared by electrostatic complexation of single-stranded oligonucleotides (6mer, 14mer, 22mer and 50mer) with a single type of cationic surfactant (dioctyldimethylammonium bromide (DOAB), didecyldimethylammonium bromide (DEAB) and didodecyldimethylammonium bromide (DDAB)) or with a two-component mixture of surfactants (DOAB+DEAB and DEAB+DDAB). DNA–surfactant LCs were obtained according to a previously published protocol^33^ that requires precipitating the DNA out of solution using the cationic surfactant, extracting the solid product by centrifugation, and lyophilizing the complex to remove any excess water. The resulting product exhibits discrete phase transitions from the isotropic liquid, to the LC and crystalline phases at temperatures characteristic of the material. For example, the DNA–DOAB complex exhibits a smectic A LC phase between −7 and 41 °C (Fig. 1 and Supplementary Fig. 1). In this lamellar LC phase, each repeating layer consists of a cationic surfactant bilayer that electrostatically interacts with an anionic oligonucleotide sublayer^33^. Within the DNA sublayer, the single-stranded oligonucleotide chains are randomly oriented, without any positional or orientational order. Long-range periodic layer structures in the LC phase have been directly visualized by freeze-fracture transmission electron microscopy (Supplementary Fig. 2). The degree of crystallinity was also investigated by wide-angle X-ray scattering. When the DNA–DOAB sample is heated above 41 °C, the X-ray scattering peak that was observed in the LC phase disappears, indicating a transition to the disordered liquid state (Supplementary Fig. 1b). If the sample is cooled below −7 °C, a series of sharp, high-intensity diffraction peaks provide evidence of crystallinity (Supplementary Fig. 1d). The phase transition temperatures of DNA–surfactant complexes can be controlled over a wide temperature range by varying the alkyl chain length of surfactants and using mixtures of surfactants complexed to the DNA (Supplementary Fig. 3). To study the optoelectronic behaviour of the samples, an LC cell was used that consists of two indium tin oxide (ITO)-coated glass plates separated by a gap of ∼6.8 μm. The LC cell was filled with the sample in the isotropic state by capillary action. When the samples were cooled to the smectic LC phase, focal-conic domains were observed by polarizing optical microscopy (POM; Fig. 1c). In these homogenous alignments, the smectic layers of the DNA–surfactant LC were oriented perpendicular to the electrode surface (Fig. 1d).

### Electrochemical properties of DNA–surfactant LCs

**Figure 2:**
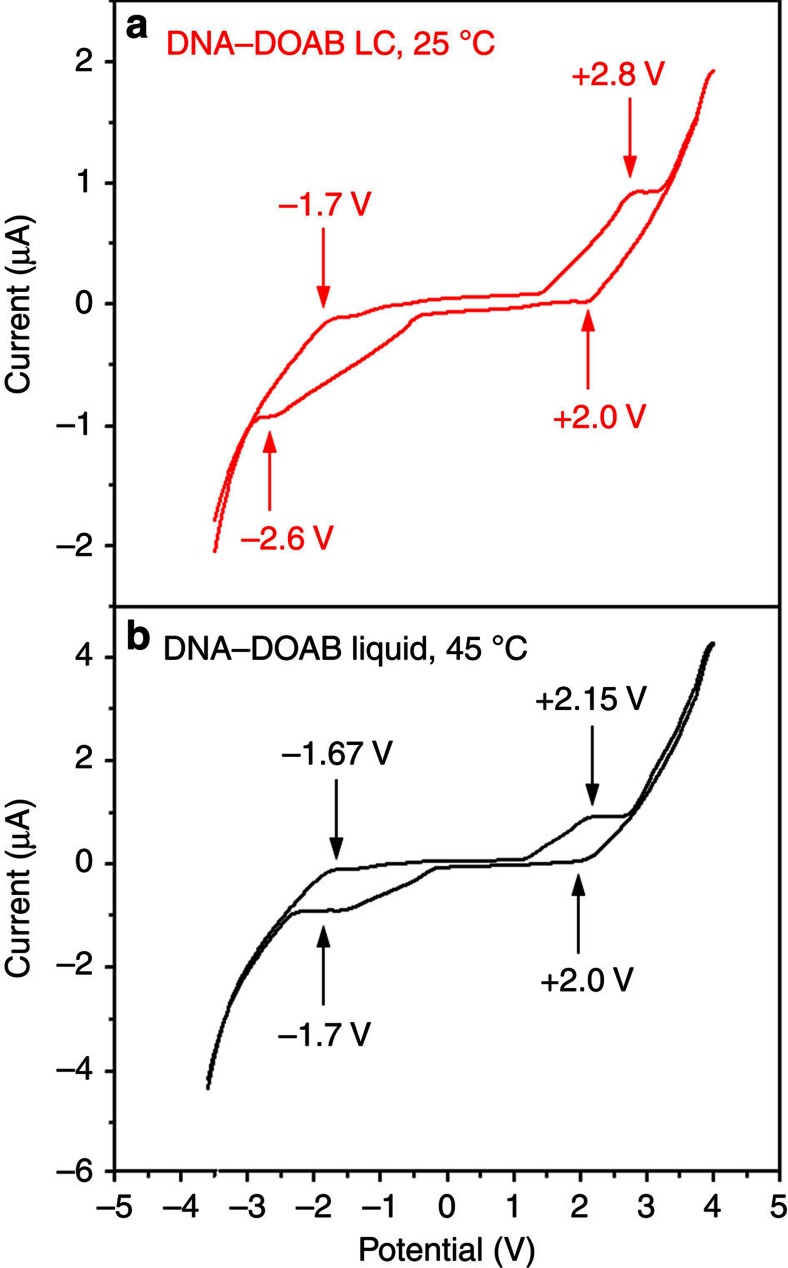
Bulk cyclic voltammetry spectra of the DNA–surfactant LC material. Here taking 14mer DNA–DOAB as an example, the DNA–DOAB liquid crystal was filled into a two-ITO-electrode LC cell by capillary force (without reference electrode). In smectic LC phase (**a** red curve, 25 °C), reversible anodic oxidation and cathodic reduction processes of nucleobases were observed at half-wave potentials of +2.4 and −2.15 V, respectively. In isotropic liquid phase (**b** black curve, 45 °C), similar redox behaviours were observed, but at lower half-wave potentials (+2.07, –1.68 V).

The redox properties of the DNA–surfactant materials were examined by cyclic voltammetry measurements. In the bulk LC state, the electrochemical spectrum of the DNA–surfactant complex was acquired in a two-ITO-electrode LC cell at 25 °C, where direct current (d.c.) electric field was applied in the direction of the DNA–surfactant smectic layers (Fig. 2, red curve). Indeed, reversible anodic oxidation and cathodic reduction processes of DNA were observed at the half-wave potentials of +2.4 and −2.15 V, respectively. Increasing the temperature to the isotropic phase of the LC material (Fig. 2, black curve), similar redox behaviours were observed, but at lower half-wave potentials of +2.07 and −1.68 V. This indicates that the activation of nucleobase in the isotropic phase is more efficient due to the higher molecular mobility than in the LC phase. The redox behaviour of the DNA–surfactant complex was also investigated in CH_2_Cl_2_ solution (Supplementary Fig. 4). Reversible anodic oxidation at the potential of+1.8 V versus Ag^+^/Ag was detected. Moreover, a pristine surfactant like DOAB did not show any redox properties.

### Switchable electrochromic behaviour in the isotropic liquid phase

**Figure 3:**
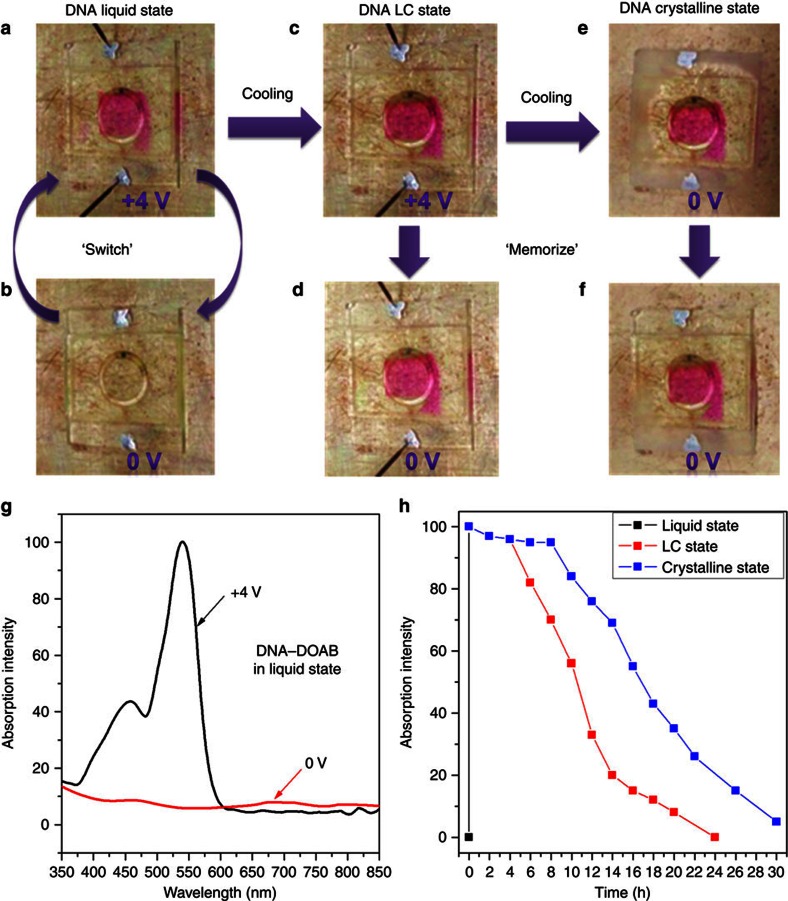
Phase-dependent electrochromism of DNA–surfactant complex in a LC cell. Here using the 14mer DNA–DOAB complex as a representative example. The ring in the images is a part of the hot stage, which is affixed to the cell by vacuum. (**a**,**b**) Switchable electrochromism between the coloured (magenta) and colourless states occurred at the anode in the isotropic liquid phase (45 °C, switch time ∼30 s). (**c**) When the coloured DNA–DOAB liquid was cooled to the LC phase (25 °C, cooling rate 5 °C min^−1^) in the presence of applied voltage, the colouration state was preserved. (**d**) When the bias was removed, remarkable optical memory of the DNA–DOAB smectic LC can be observed as a persistent coloured state. (**e**) Further cooling the coloured DNA–DOAB LC phase to the crystalline phase (−20 °C) without the application of voltage, (**f**) the relaxation rate of the coloured state was significantly reduced. (**g**) Strong absorption bands in the ultraviolet–visible absorption spectrum of the DNA–DOAB liquid (45 °C) appeared in the region of 350 to 600 nm under applied voltage (black curve) and disappear shortly after the voltage was removed. (**h**) Decay kinetics of the coloured state in different phases (the points were recorded after the applied voltage was returned to 0 V). In the isotropic liquid phase, the colour decayed completely within ∼30 s after a removal of bias (black curve). In the smectic LC phase (red curve, corresponding to Fig. 2d), the colour intensity was maintained above 95% for 4 h and the colourless state was recovered within 24 h. In the crystalline phase (blue curve, corresponding to Fig. 2e,f), the colour intensity is maintained above 95% for ∼8 h and complete bleaching was only evident at 30 h.

The DNA–surfactant complex exhibits reversible electrochromic switching in the isotropic liquid phase in the absence of liquid electrolyte (Fig. 3). A d.c. voltage step from 0 to 4 V causes the DNA–DOAB material to change colour from clear to magenta at the anode of the cell (Fig. 3a,b). This gave rise to an absorption in the region from 350 to 600 nm (Fig. 3g). In view of the redox behaviour analysis of the complex, this suggests that DNA in the bulk and solution states undergoes reversible anodic oxidation when a positive potential is applied (Fig. 2 and Supplementary Fig. 4). The oxidized state of DNA in CH_2_Cl_2_ solution also produced an absorption between 350 and 600 nm (Supplementary Fig. 5). These results are in agreement with oxidation experiments involving radical cations of nucleobases^35^,^36^,^37^,^38^,^39^, which is accompanied by the same ultraviolet–visible absorption^35^,^36^,^37^. Thus, these observations for the solution phases suggested that the colour change of the DNA–surfactant complex from colourless to magenta (350–600 nm absorption) in the bulk state can be attributed to the formation of nucleobase radical cations at the LC cell anode. The transparent state can then be recovered within several seconds when the voltage across the electrochromic cell is returned to 0 V. Thus, the DNA–DOAB complex exhibits reversible electrochromic properties in the isotropic liquid phase. Control experiments showed that such colour changes did not occur in the pristine surfactant material that lacks a redox-active moiety. Thus, we determine that the solvent-free DNA–surfactant complex can be considered the first example of DNA electrochromism. Previously, DNA has only acted as an electrolyte or as the host for dispersing electrochromic materials in electrochromic devices^40^,^41^.

Optical switching rates of the DNA–surfactant complexes in the isotropic phase were examined by applying a double potential step from 0 to 4 V and back to 0 V. It is found that the electrochromic response time of these materials is correlated with the length of the DNA used (Table 1 and Supplementary Fig. 6), suggesting that the rate of DNA oxidation is limited by the rate of mass transport to the anode. The 6mer DNA–DOAB liquid exhibited response time of about 15 s in switching between the coloured and colourless states, whereas the 14mer, 22mer and 50mer had significantly longer response times of 30, 80 and 120 s, respectively. These results indicate that increasing the dynamic radius of the DNA component leads to a reduction in the rate of DNA diffusion and thus affects the molecular mobility of the chromogenic component. To investigate the electrochromic stability of these DNA–surfactant ionic materials, 30 cycles of colouration and bleaching were carried out in a sequence of double-potential steps (Supplementary Fig. 7a, last seven cycles shown for 6mer DNA–DOAB; Supplementary Fig. 7c, last two cycles shown for 22mer DNA–DOAB). The colour switching remained reversible and switching time of the last cycle did not significantly deviate from that of the first cycle (Supplementary Fig. 6). Homogeneous focal-conic textures were recovered after cooling the electrochemically cycled samples to the LC phase (Supplementary Fig. 7b,d), indicating that the ionic DNA–surfactant complexes are stable within this time frame.

### Optical memory behaviours in the LC and crystalline phases

When the DNA–surfactant liquid was cooled to the smectic LC phase while an applied voltage of 4 V was maintained, the magenta colour was conserved (Fig. 3c). Even after the cell voltage was returned to 0 V, the colouration state was temporally preserved in the smectic LC phase (Fig. 3d) and completely bleached within 24 h (Fig. 3h, red curve and Supplementary Fig. 8). Further cooling the DNA–DOAB material in the magenta colour state from the LC to the crystalline phase in the absence of applied voltage (Fig. 3e,f) extended the persistence time of the magenta state (Fig. 3h, blue curve and Supplementary Fig. 9). The complete recovery of the colourless state in the crystalline phase was observed within about 30 h. In addition, the coloured DNA–DOAB was bleached immediately after extracting the material from the cell by ethanol (Supplementary Fig. 10). This suggests that the radical cations are reduced quickly in solution. These results clearly indicated that the radical cations of nucleobases can be stabilized in the solvent-free and structured DNA–surfactant materials. Thus, a method to modulate the volatility of the optical memory was developed by controlling the phase of the DNA–surfactant complex.

**Figure 4:**
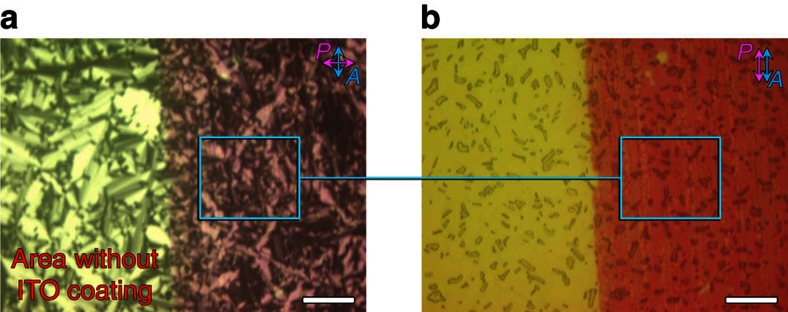
The study of electrochromic behaviours under microscopy. Investigation of birefringence textures, orientation and colour domains when the DNA–surfactant liquid was cooled to the smectic LC phase while an applied voltage of 4 V was maintained (here 14mer DNA–DOAB complex as a representative example). The image of the coloured DNA–DOAB textures obtained with crossed polarizer and analyzer (**a**) and the corresponding image (**b**) where the polarizer and analyzer were parallel. Note that the left part of the images represents areas of the cell without conducting ITO coating, whereas the right parts shows the conductive ITO electrode. The DNA–DOAB formed a homogenous coloured film. The focal-conic textures became much smaller or disappeared in the coloured DNA–DOAB domains when compared with the uncoloured birefringence domains. Besides a largely perpendicular lamellar orientation (DNA–DOAB layers oriented vertical to electrode surface), reorientation of the oxidized DNA smectic layers took place, adopting a new type of horizontal alignment (DNA–DOAB layers oriented parallel to electrode surface). Scale bar, 100 μm.

**Figure 5:**
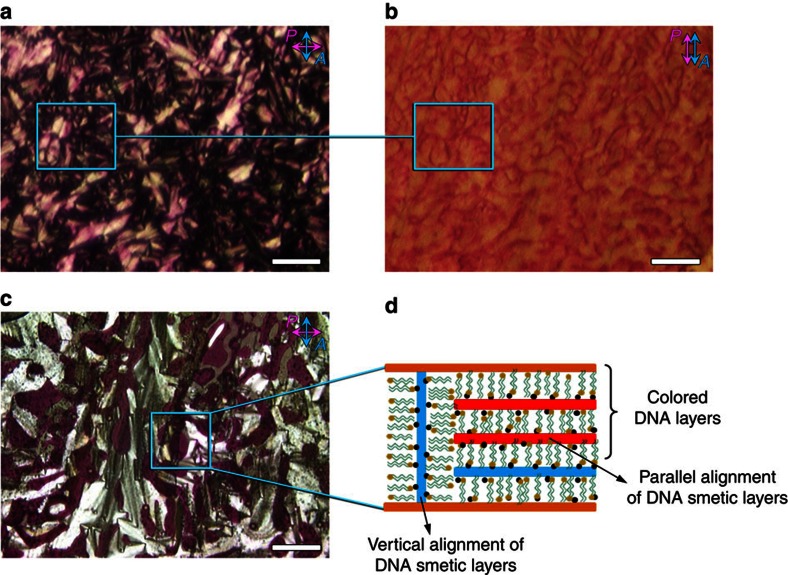
The study of electrochromic behaviours under microscopy. Investigation of birefringence textures, orientation and colour domains of the coloured DNA–surfactant complex in the LC state (25 °C) in the absence of an applied voltage (corresponding to Fig. 2d, 14mer DNA–DOAB with almost no colour decay within 4 h). The image of the coloured DNA–DOAB textures obtained with crossed polarizer and analyzer (**a**) and the corresponding image (**b**) where the polarizer and analyzer were parallel. The coloured DNA within normal perpendicular lamellar orientation was bleached, whereas the horizontal alignment of DNA–surfactant layers preserved the colouration state. (**c**) This reorientation effect became more apparent after 10 cycles of applying a positive potential in the isotropic state, cooling to the LC state, removing the potential and again heating to the isotropic state. The coloured DNA–DOAB smectic layers oriented parallel to the electrode surface, whereas the bleached DNA–DOAB layers remained vertical to the electrode surface. (**d**) The corresponding sketch of the lamellar alignment of the coloured and the bleached DNA–DOAB domains. Scale bar, 50 μm.

POM was used to investigate the birefringence textures and they relate to the activated colour domains. In the isotropic liquid and smectic LC phases with an applied voltage of 4 V, a homogenous formation of red colour was observed in the device (Fig. 4 and Supplementary Fig. 11). It is found that the focal-conic textures became much smaller or disappeared in the coloured DNA–DOAB domains in comparison with the uncoloured DNA–DOAB birefringence domains without ITO coating (Fig. 4). We assume that reorientation of the oxidized DNA–DOAB smectic layers took place due to the applied voltage during the transition process from the isotropic to the LC phase (Fig. 3, from a to c). In the presence of an applied voltage, the capacitive positive charge at the anode may attract the negatively charged DNA, favouring a horizontal alignment of the nucleic acid layers in relation to the anode surface. When the applied voltage was switched to 0 V, the coloured DNA with perpendicular lamellar orientation was bleached (Fig. 5). However, the horizontally aligned DNA–surfactant layers preserved the colouration state. This reorientation effect (compare Figs 1d and 5d) became more apparent after many cycles of applying a positive potential in the isotropic state, cooling to the LC state, removing the potential and again heating to the isotropic state (Fig. 5c,d). The coloured DNA–DOAB smectic layers oriented parallel to the electrode surface, whereas the bleached DNA–DOAB layers remained vertical to the electrode surface. Furthermore, the alignment of the DNA–DOAB lamellar layers was maintained after the colour decays completely (Supplementary Fig. 12a–c). The perpendicular orientation of the bleached DNA–DOAB smectic layers was recovered after an annealing treatment without application of voltage (Supplementary Fig. 12d), confirming the integrity of the ionic DNA–DOAB complexes. Further cooling of the coloured LC sample to the crystalline phase preserved the reorientation of oxidized DNA–DOAB smectic layers (Supplementary Figs 13 and 14). The above results suggest that the reorientation of the oxidized DNA–DOAB smectic layers played an important role in trapping and protecting the coloured radical cations and thus, in achieving the storage characteristics of optical information in the LC and crystalline states.

**Figure 6:**
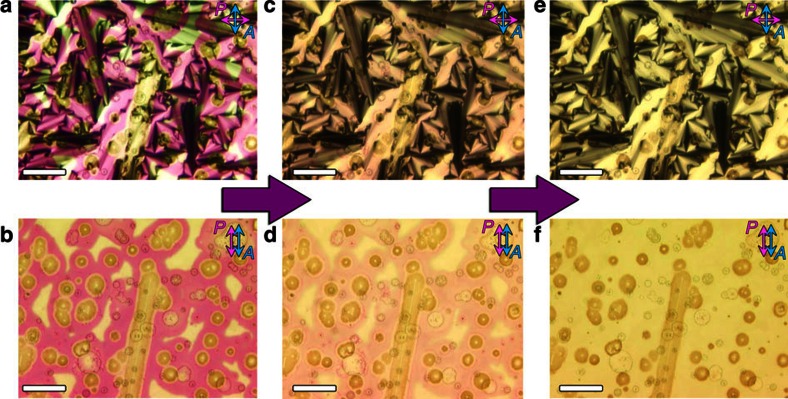
The study of electrochromic behaviours under microscopy. Investigation of birefringence textures, orientation, colour domains and colour decay after the application of 4 V directly to a DNA–DOAB complex in the LC phase (scale bar, 100 μm). The optical images between crossed polarizer and analyzer (**a**, 0 h; **c**, 2 h; **e**, 4 h) and the corresponding images with parallel polarizer and analyzer (**b**, 0 h; **d**, 2 h; **f**, 4 h) showed that the birefringence domains of coloured DNA–DOAB smectic layers remained unaltered, exhibiting perpendicular lamellar orientation. The colourless state was recovered in ∼3 h, which was a significantly shorter recovery time than when the coloured LC state was obtained from the isotropic phase.

During a control experiment, when a potential of 4 V was applied directly to the DNA–DOAB material in the LC phase at 25 °C, the colour impression appeared much slower and with lower intensity than in the isotropic liquid phase (Supplementary Fig. 15). This can be attributed to the lower mobility of DNA molecules in the smectic LC phase. In contrast to the colouration attained in the isotropic phase before transition to the LC phase, the colouration attained in the LC phase decayed completely within 3 h (Supplementary Fig. 15), indicating that the relaxation time of DNA radical cations was significantly shorter than in the coloured LC sample annealed from the isotropic phase (Fig. 3d,h, red curve). Interestingly, POM analysis did not reveal a difference in the alignments of coloured and uncoloured DNA–DOAB domains, as both exhibited perpendicular orientation of the smectic layers on the ITO surface (Fig. 6).

### Mechanism for phase-dependent DNA electrochromism

**Figure 7:**
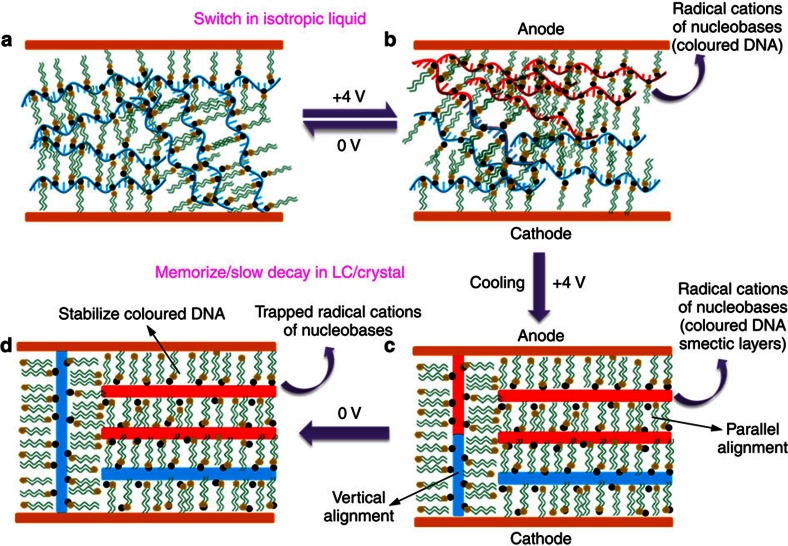
Electrochromic mechanism of the DNA–surfactant complexes. (**a**) The fluid DNA–surfactant material in their isotropic liquid phase. (**b**) Upon applying an electric field, reversible anodic oxidation and cathodic reduction processes of nucleobases take place. Radical cations are produced on the anodic electrode surface, resulting in the colouration of the DNA–surfactant materials. Once a potential of 0 V is applied, the radical cations are reduced and the bleaching process takes place. Thus, switchable electrochromic response is observed in the isotropic liquid phase. (**c**) When the complex is cooled to the smectic LC or crystalline phases under applied voltage, the coloured DNA–surfactant complexes develop a horizontal lamellar alignment besides the normal perpendicular lamellar orientation. (**d**) When the applied voltage is returned to 0 V, the coloured DNA within perpendicular lamellar orientation is bleached. The horizontal alignment of DNA–surfactant layers preserved the colouration state, which is responsible for trapping and protecting the coloured radical cations and thus, in achieving the optical storage characteristics in the LC and crystalline states.

As depicted in Fig. 7a,b, in the isotropic phase, mobile segments of oligonucleotide are oxidized at the anodic electrode when a positive potential of 4 V is applied. The accumulation of radical cations manifests as the magenta coloured state. When a potential of 0 V is applied, the DNA radical cations are reduced, which leads to rapid bleaching in the liquid state. When the coloured DNA–surfactant liquid is cooled while a positive potential of 4 V is applied, the magenta DNA–surfactant complexes develop a lamellar LC structure with parallel alignment to the electrode surfaces besides the normal perpendicular lamellar orientation (Fig. 7c). When the electric field is switched off, the coloured DNA with perpendicular lamellar orientation is bleached (Fig. 7d). This is probably due to the efficient electron transfer through vertical nucleobase sublayers. However, in the horizontal alignment of the oxidized DNA–surfactant complex, the surfactant sublayers may act as insulating barrier and prevent electron hopping. Therefore, the reduction process of DNA radical cations might be slowed down and the generated colouration state could be preserved long time (Fig. 7d). It should be also noted that the oxidized DNA molecules diffuse further away from the anode surface in the isotropic phase than in the LC state. Therefore, in cooled samples from the isotropic melts, the DNA radical cations are trapped and the coloured state requires a longer time to relax back to the colourless state compared with samples that are directly oxidized in the LC state. Owing to the mechanisms discussed above, the response of a coloured state of DNA–surfactant material can be controlled via a simple phase transition strategy in the absence of applied voltage.

### Tunability of optical memory performance

**Figure 8:**
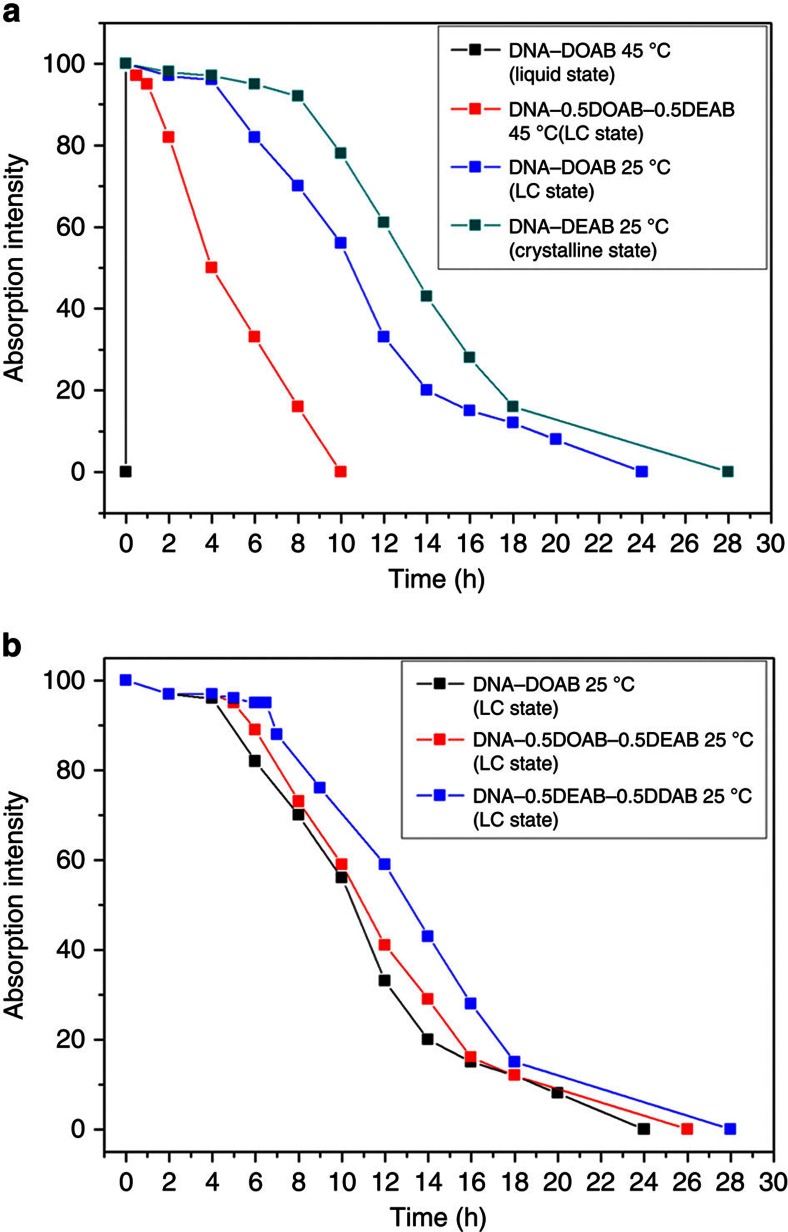
Colour decay kinetics of the DNA–surfactant samples. The materials are complexed with different surfactants, surfactant mixtures and 14mer DNA. (**a**) Phase-dependent memory behaviour of the materials. The magenta colour of DNA–DOAB isotropic liquid was bleached in 30 s at 45 °C after removal of bias (black curve). At the same temperature, the colour impression of the DNA–0.5DOAB–0.5DEAB smectic LC phase was preserved almost completely for ∼1 h after the removal of the bias (black curve). At 25 °C, the DNA–DOAB smectic LC exhibited enhanced optical memory with negligible colour decay within ∼4 h (blue curve). The crystalline state of the DNA–DEAB at this temperature showed a much longer relaxation time of ∼7.5 h (green curve). (**b**) Viscosity-dependent memory behaviours of the materials. The magenta colour impression of the DNA–surfactant smectic LCs (25 °C) was extended from ∼4 h (DNA–DOAB, black curve) to ∼5.5 h (DNA–0.5DOAB–0.5DEAB, red curve) and further to ∼7 h (DNA–0.5DEAB–0.5DDAB, blue curve) with increasing the alkyl chain lengths of the surfactants.

To further illustrate the importance of material phases (isotropic liquid–LC–crystal) for tuning durability of stored optical information, a series of DNA–surfactant complexes with phase transition temperatures that range from −20 to 130 °C have been prepared (Supplementary Fig. 3). It can be seen that the magenta colour of the DNA–DOAB liquid decays within 30 s after removing the potential at 45 °C (Fig. 8a, black curve and Supplementary Fig. 6b). At the same temperature, DNA–0.5DOAB–0.5DEAB adopted the smectic LC phase and the colour impression, after activation in the isotropic state, is decaying within ∼10 h (Fig. 8a, red curve and Supplementary Fig. 16). This indicates that the lamellar layers of the DNA–surfactant complex can trap and stabilize the radical cations much more efficiently than the disordered liquid phase. At 25 °C, the DNA–DOAB smectic LC exhibited an extended optical memory, showing full colour decay within ∼24 h after removal of applied potential (Fig. 8a, blue curve and Supplementary Fig. 8). The crystalline state of the DNA–DEAB at this temperature showed a longer memory time of ∼28 h until full bleaching occurred (Fig. 8a, green curve and Supplementary Fig. 17). We attribute the differences in the rates of colour formation and decay among the different phases and surfactant compositions to differences in the ionic mobility of the coloured DNA component. We have previously reported that the viscosity of the DNA–surfactant complex, which was inversely proportional to ionic mobility, increased with the increasing surfactant alkyl chain length^34^. It is thus reasonable that the rate of decay of the magenta colour impression correlated inversely with LC viscosity (Fig. 8b and Supplementary Figs 18 and 19).

### DNA-LC optical memory device for smart tag application

**Figure 9:**
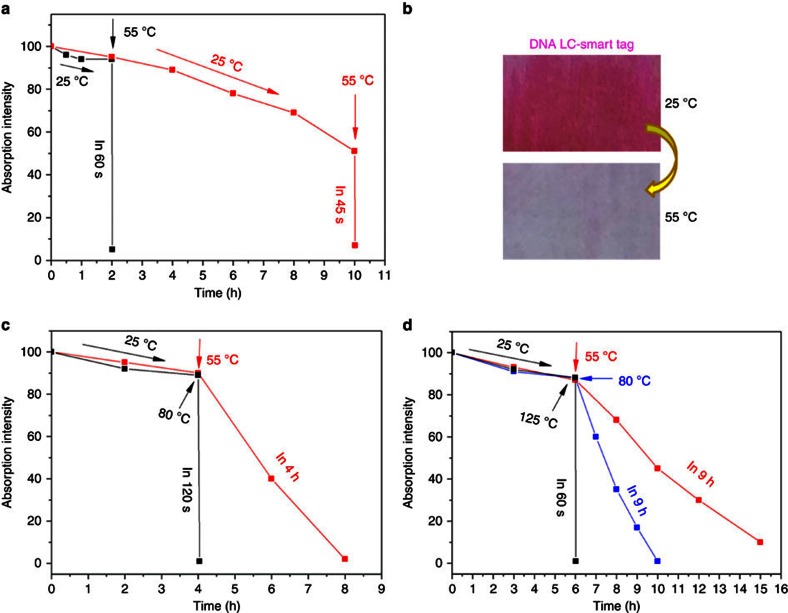
Decay kinetics of the coloured DNA–surfactant samples. (**a**) At 25 °C, the color-activated 14mer DNA–DOAB LC sample showed a very slow colour decay in the absence of applied voltage. Once the sample was heated to 55 °C (for example, after 2 or 10 h after activation of the memory device), the colour decayed completely within 60 s. (**b**) The corresponding photographs of the coloured (25 °C) and bleached (55 °C) samples. (**c**) For activated 14mer DNA–0.3DOAB–0.7DEAB at 55 °C, the bleaching process is significantly slowed down, whereas at 80 °C the colour decay is realized within 2 min. (**d**) For activated 14mer DNA–0.3DEAB–0.7DDAB, a temperature increase to 55 and 80 °C yields only a slow colour decay, but above a temperature of 125 °C, bleaching occurs within a minute.

Two important features of the electrochromic behaviour of DNA–surfactant complexes have been demonstrated above. (1) The decay time of the coloured state in the isotropic melt is in the range of seconds depending slightly on the length of the DNA molecule. (2) The decay in the LC state occurs within several hours and can be tuned by the selection of the surfactant complexed with DNA. These intriguing material characteristics can be combined and exploited for the fabrication of smart tags that exhibit a clock function and a ceiling temperature indicator. For demonstration of this dual functionality, we have chosen the 14mer DNA–DOAB complex as an example. After activation of the tag in the isotropic melt, cooling and removal of voltage, the coloured state decayed slowly at room temperature. However, when the temperature was raised to 55 °C, after 2 h, the magenta colour disappeared within 60 s (Fig. 9a, black curve and Fig. 9b; Supplementary Fig. 20). Similar, a sample with ∼50% colour decay after 10 h was bleached within 45 s when the temperature was adjusted to 55 °C (Fig. 9a, red curve; and Supplementary Fig. 21). Taking into account that the isotropic liquid–LC transition in the DNA–surfactant materials can be conveniently tuned in the wide range between 130 and 41 °C (Supplementary Fig. 3), a series of smart tags with different decay behaviours and ceiling temperatures can be fabricated. This is illustrated in Fig. 9c (Supplementary Fig. 22) and Fig. 9d (Supplementary Fig. 23) for DNA–0.3DOAB–0.7DEAB and DNA–0.3DEAB–0.7DDAB, respectively. Although for the former material an instantaneous colour decay is observed at already 80 °C, the latter DNA–surfactant complex requires a temperature of 125 °C for bleaching in seconds. One can imagine that these behaviours of DNA–surfactant LCs offer great opportunities for developing smart tag applications, especially with respect to packed perishable product inspection. We equipped a food package with an activated DNA-LC device and demonstrated functionality as combined time and temperature indicator (Supplementary Fig. 24).

## Discussion

A new type of electrochromic material based on redox-active nucleic acids has been developed by mixing DNA and cationic surfactant. Phase-dependent electrochromism is an inherent characteristic of the DNA–surfactant complex, where fluidity and ordering are introduced by ionic self-assembly^42^,^43^,^44^. The rate of colour decay was modulated very broadly, simply by controlling the phase of the material. In the isotropic liquid phase, the DNA–surfactant complex exhibits stable, reversible switching between the colourless and magenta states. In stark contrast to conventional LC electrochromic devices^7^,^24^,^25^,^26^, the colouration state can be maintained over an extended period of time in the smectic LC phase or the crystalline phase without applying voltage. The time required for complete decay can range from a few seconds to over a day, which provides for a simple and effective way to modulate the volatility of stored optical information. This new type of DNA materials can be easily processed to fabricate single-layer electrochromic devices without the need for external electrolyte layers. To the best of our knowledge, the approach to controlling the volatility of stored optoelectronic information by modulating the phase of the material has never previously been proposed or attempted. Due to the broad and stable phases, these DNA–surfactant materials hold great promise for the realization of novel types of smart tags or optical devices as well as for diagnostic devices where recognition or biocatalytic events lead to phase transitions.

## Methods

### Materials

The surfactants used for the DNA complex formation, including DOAB and DDAB were purchased from ABCR (Germany), and DEAB was aquired from Sigma Aldrich. Single-stranded DNA, including 6mer (5′-CCTCGC-3′, molecular weight (MW)=1,728 g mol^−1^), 14mer (5′-CCTCGCTCTGCTAA-3′, MW=4,175 g mol^−1^) and 22mer (5′-CCTCGCTCTGCTAATCCTGTTA-3′, MW=6,612 g mol^−1^) were synthesized by conventional solid-phase synthesis method^45^. The 50mer DNA strand (5′-CCTCGCTCTGCTAATCCTGTTACCTCGCTCTGCTAATCCTGTTACCTCGC-3′, MW=15,077 g mol^−1^) was purchased from BIOMERS (Germany). Tetrabutylammonium perchlorate (Bu_4_NClO_4_, Mw=341.91 g mol^−1^), used as the supporting electrolyte for cyclic voltammetry measurements was purchased from Sigma.

### DNA–surfactant complex preparation

The single-stranded DNA (6mer, 14mer, 22mer and 50mer) was pretreated by precipitating a ∼300 μM DNA solution containing 5 M NaCl with cold ethanol (−20 °C). From this precipitate, an aqueous DNA solution (∼300 μM) was prepared in ultrapure water. In a second solution made from ultrapure water, the concentration of single surfactant (DOAB, DEAB and DDAB) or a two-surfactant mixture (DOAB:DEAB, DOAB:DDAB and DEAB:DDAB, molar ratios 7:3, 5:5 and 3:7) were adjusted to 5–10 mM at room temperature. Both the DNA and surfactant solutions (∼5 mol equivalents of surfactant relative to nucleotides of the DNA) were mixed together and as a result the insoluble complexes precipitated from the aqueous phase. After centrifugation, the water and unreacted surfactants were removed, and finally the complexes were lyophilized overnight before further characterization.

### Electrochromic device fabrication

The prepared DNA–surfactant complexes were filled into a commercial liquid crystal cell consisting of two ITO-coated glass plates (LC4–6.8, ITO area 5 mm × 5 mm, gap 6.8 μm, INSTEC, USA). There are two openings on the cell. A small amount of the DNA–surfactant sample was placed over one opening, which lead to capillary forces pulling the liquid crystal material into the empty cell. This process can be accelerated by heating the materials to the isotropic states.

### Electrochromic measurement

The DNA–surfactant complex was introduced into the electrochemical cell and heated to the isotropic phase. The potential was then switched between 4 and 0 V over the course of the switching response studies. Subsequently, the complex was cooled to the smectic LC phase (cooling rate 5 °C min^−1^) under an applied potential of 4 V. After cooling, the electric field was switched off to study the relaxation time of the coloured state. In other experiments, the coloured LC state was immediately cooled to the polycrystalline phase (cooling rate 5 °C min^−1^) in the absence of an applied electric field.

### Characterization of DNA–surfactant liquid crystals

Thermal behaviours of the DNA–surfactant complexes were investigated by differential scanning calorimetry using a TA Instruments Q1000 system in a nitrogen atmosphere with a heating/cooling rate of 5 °C min^−1^. The structural features of the DNA–surfactant complexes were analysed by wide-angle X-ray scattering (WAXS). WAXS with heating and cooling systems was carried out by a custom, rotating-anode-based setup^46^, made in-house, where the sample-to-detector distance was 13 cm and a conventional X-ray source with radiation wavelength of *λ*=1.54 Å was used. Smectic layer structures of the DNA–surfactant complexes were carried out by freeze-fractured transmission electron microscopy (FF-TEM) according to a standard protocol^47^. FF-TEM was performed by sandwiching the samples between 2 mm by 3 mm glass planchettes and cooling from the isotropic melt to a selected temperature in the LC range. The samples were then rapidly quenched to T<−180 °C by immersion in liquid propane, fractured in vacuum at −140 °C and then coated with 2 nm of platinum and then with 25 nm of carbon. After dissolving the liquid crystal, the Pt–C replicas were placed in the TEM, where the topographic structure of the fracture plane was observed. Textures and orientation analysis of the complexes were studied by POM. POM was conducted on a Zeiss Axiophot using the same temperature program that was used for the differential scanning calorimetry experiments. Electrochemical behaviours of the DNA–surfactant complexes were investigated in the solution and in the bulk material. Standard cyclic voltammetry (CV) in solution was performed in a three-electrode cell equipped with a Pt working electrode, a Pt counter electrode and an Ag^+^/Ag reference electrode. A solution of Bu_4_NClO_4_ in CH_2_Cl_2_ (0.10 M) was used as the supporting electrolyte. The sweep rate is 100 mV s^−1^. The electrical spectrum in the bulk (in the electrochromic cell described above) was measured by a semiconductor parameter analyzer (4155B, Hewlett packard). Electrochromic behaviours were examined on a hot stage (heating/cooling rate of 5 °C min^−1^) with d.c. voltage control. Ultraviolet–visible absorption spectra were recorded with a JASCO V-630 spectrophotometer for the study of the reversible electrochromism and optical memory (decay kinetics) properties. Electrochromic switching time between colouration and bleaching of the DNA–surfactant complexes in the isotropic liquid phases were measured and analysed by processing the recorded videos with the ImageJ software (National Institutes of Health).

## Additional information

**How to cite this article:** Liu, K. *et al*. Controlling the volatility of the written optical state in electrochromic DNA liquid crystals. *Nat. Commun.* 7:11476 doi: 10.1038/ncomms11476 (2016).

## Supplementary Material

Supplementary Information Supplementary Figures 1-24 and Supplementary References

## Figures and Tables

**Table 1:** Switching times of the DNA electrochromics in the isotropic liquid phase.

**DNA length**	**Switch on**	**Switch off**
6mer DNA	∼15 s	∼14 s
14mer DNA	∼30 s	∼26 s
22mer DNA	∼80 s	∼70 s
50mer DNA	∼120 s	>100 s

Here using the DNA–DOAB complex as a representative example (45 °C). The electrochromic response time of these materials is correlated with the length of the DNA used.
