# Supplementary material for: Controlling the volatility of the written optical state in electrochromic DNA liquid crystals
Source: Nat Commun. 2016 May 9;7:11476. doi: 10.1038/ncomms11476 (PMC4865822; doi:10.1038/ncomms11476)
Supplement: Supplementary Information — Supplementary Figures 1-24 and Supplementary References [file ncomms11476-s1.pdf]

Supplementary Figures

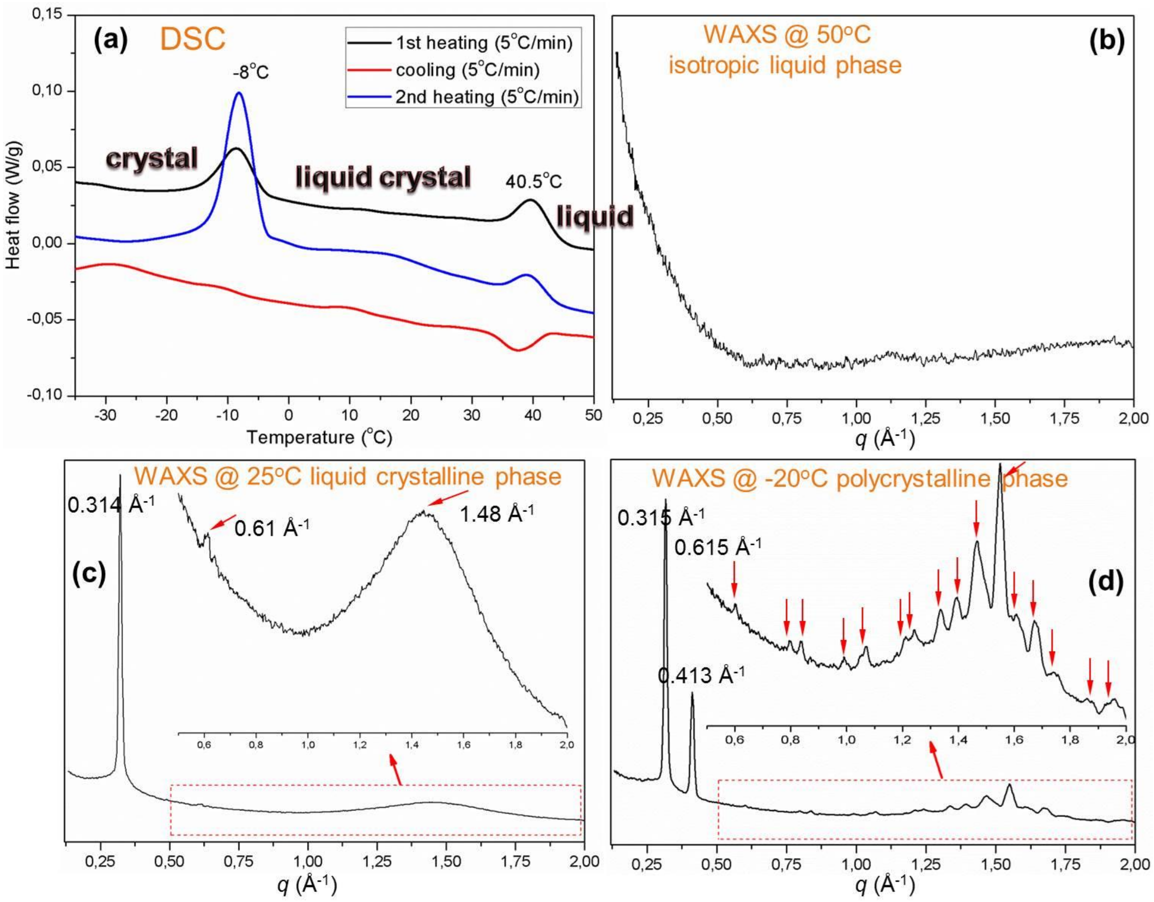

Supplementary Figure 1. Characterization of the DNA-surfactant complexes (here taking 14mer DNA-DOAB as an example). (**a**) Thermal behavior of the DNA-DOAB by differential scanning calorimetry (DSC) analysis. The DNA-DOAB exhibits continuous phase transitions from the isotropic liquid (above 41 °C) state to the liquid crystalline (from 41 to -7 °C) state to the polycrystalline state (below-7 °C). (**b-d**) Structural study of the DNA-DOAB complex by wide-angle X-ray scattering (WAXS). In the isotropic liquid state (**b**), no any X-ray diffraction peak was observed because the DNA-DOAB molecules are disordered. In the mesophase (**c**), a sharp first order peak (0.314 $Å^{-1}$) and the following harmonic (second order diffraction 0.61 $Å^{-1}$), and the diffraction halo (1.48 $Å^{-1}$) are characteristic of long-range ordered lamellar structure. In the crystalline phase (**d**), a series of diffraction peaks that are strong and narrow indicate the crystallinity of the as-prepared DNA-DOAB in -20 °C.

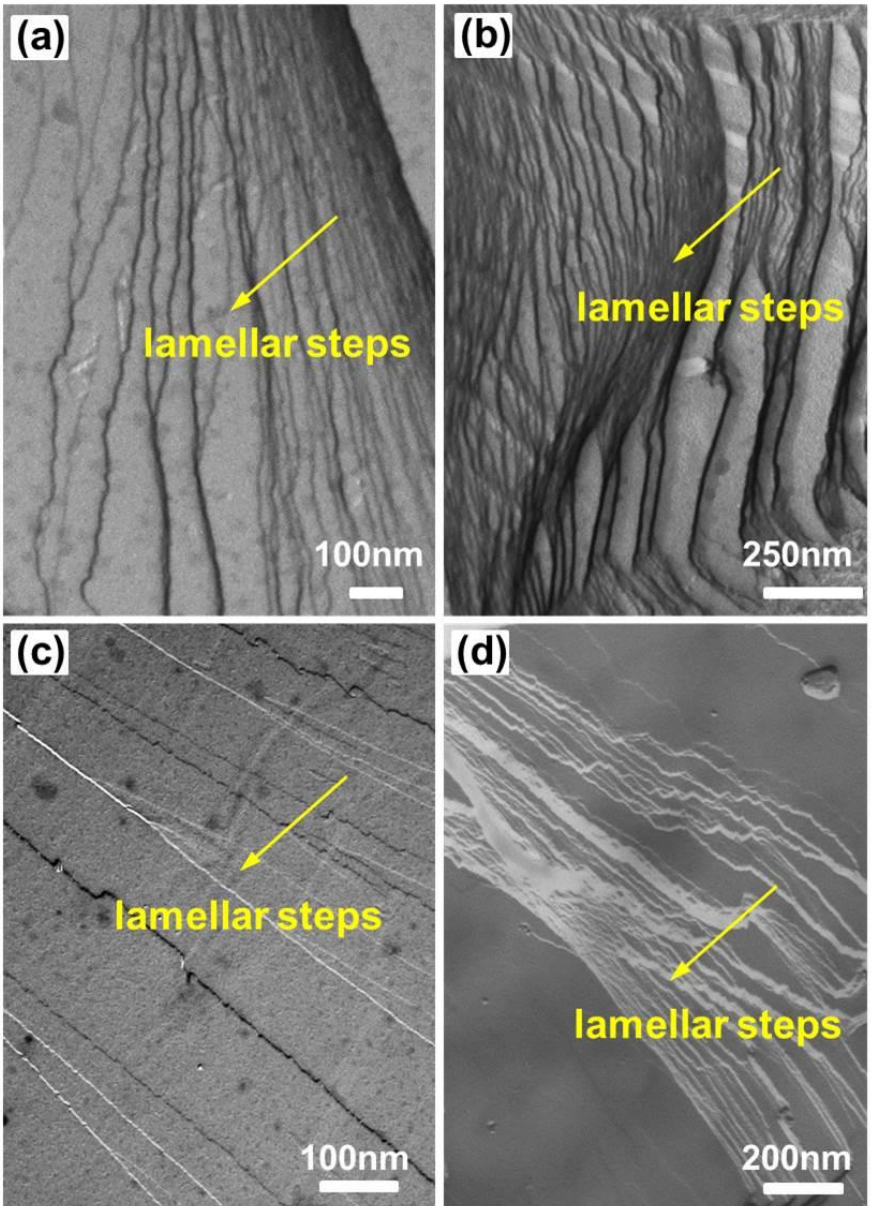

Supplementary Figure 2. The lamellar structure characterization of the DNA-surfactant mesophases by freeze-fracture transmission electron microscopy (FF-TEM). (**a**) DNA-DOAB (25°C). (**b**) DNA-DEAB (70°C). (**c**) DNA-0.5DOAB-0.5DEAB (25°C). (**d**) DNA-0.5DEAB-0.5DDAB (25°C). In all the samples, smectic layer surfaces are generally smooth but have occasional layer steps that are distinct and can be identified unambiguously.

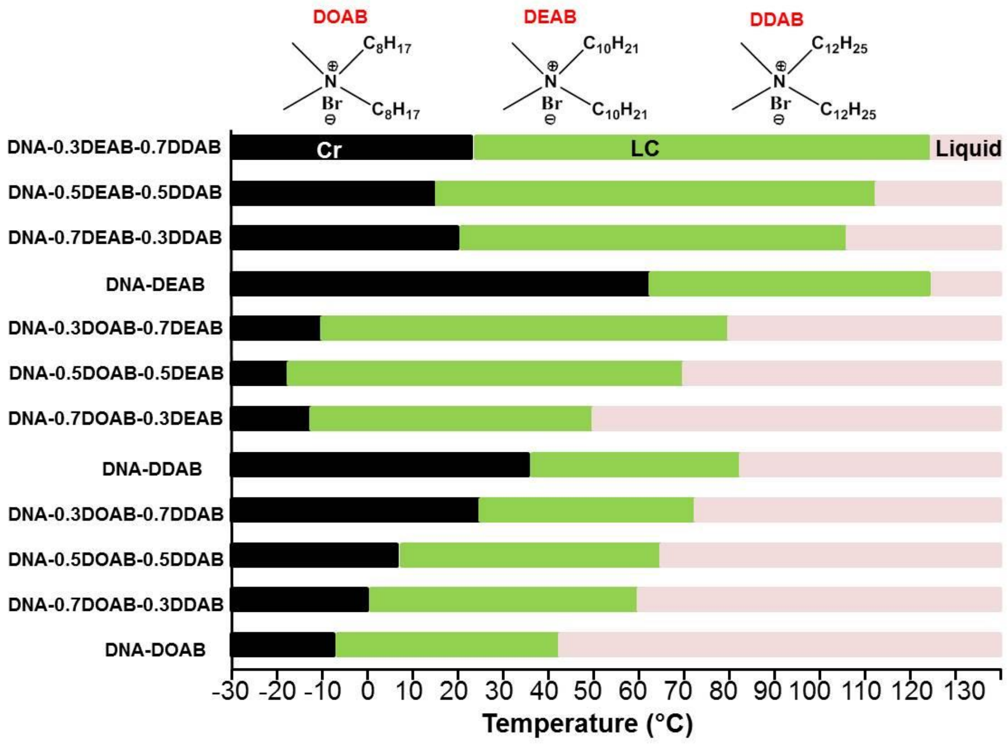

Supplementary Figure 3. Overview of thermal behaviors of binary and ternary DNA-surfactant complexes. The phase transition temperatures from crystalline (Cr) to liquid crystalline (LC) and then to isotropic liquid can be controlled over a wide temperature range. Melting temperatures are tunable from around -20°C to 65 °C, and clearing temperatures are broadly dispersed between 41 °C and ~130 °C, respectively. These behaviors are strongly dependent on the specific length of the aliphatic chains of the surfactants, and marginally influenced by the lengths of the DNA used[1].

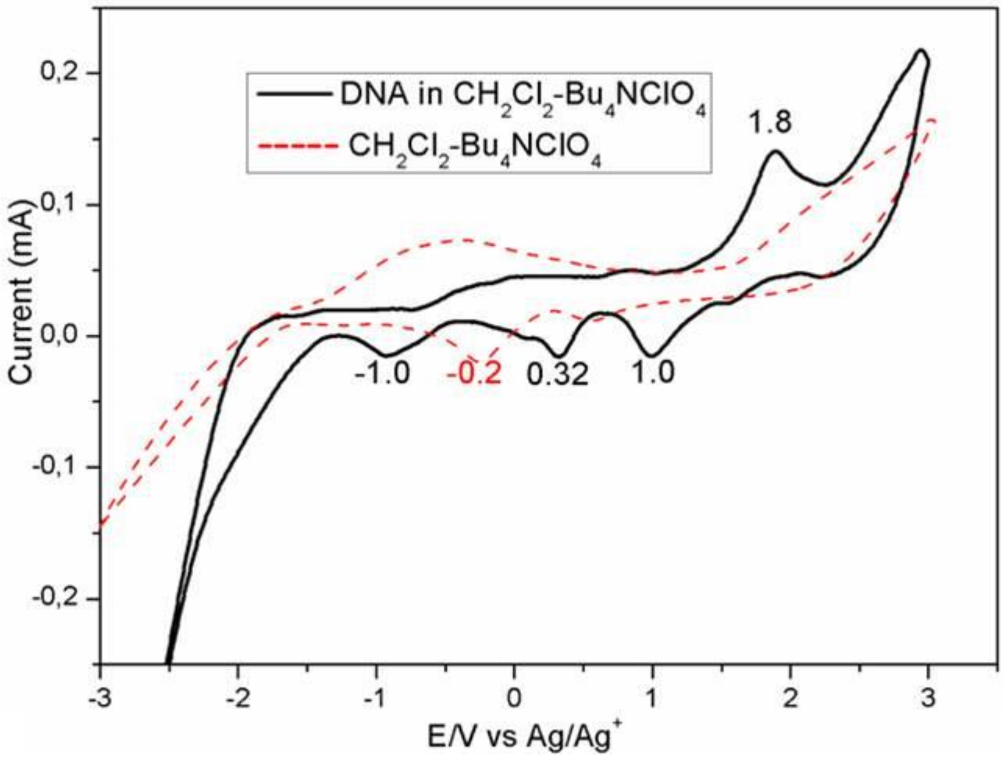

Supplementary Figure 4. Electrochemical properties of the DNA-surfactant complexes in the solution (here taking DNA-DDAB as an example, ~0.5 mM in CH_2Cl_2) investigated by standard cyclic voltammetry measurement with a Pt working electrode. Bu_4NClO_4 (0.1 M) was used as the supporting electrolyte. All potentials were measured using a Ag/AgCl electrode as a reference. Reversible anodic oxidation was observed at the potential of +1.8 V versus Ag^+/Ag.

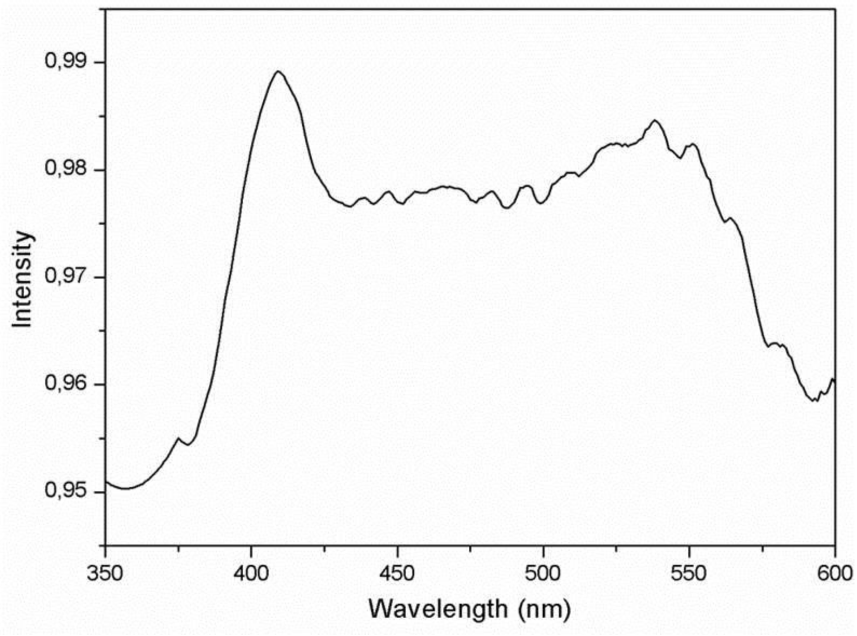

Supplementary Figure 5. Optical absorption spectrum of the DNA-DDAB complex in CH_2Cl_2 solution (DNA, ~0.5 mM; Bu_4NClO_4, ~0.1 M) with an applied potential of +2 V (Pt working electrode). Reversible anodic oxidation of nucleobases took place and gave rise to absorption in the region from 350 to 600 nm. This optical behavior is in agreement with oxidization experiments involving nucleobases and their radical cation formation[2-3].

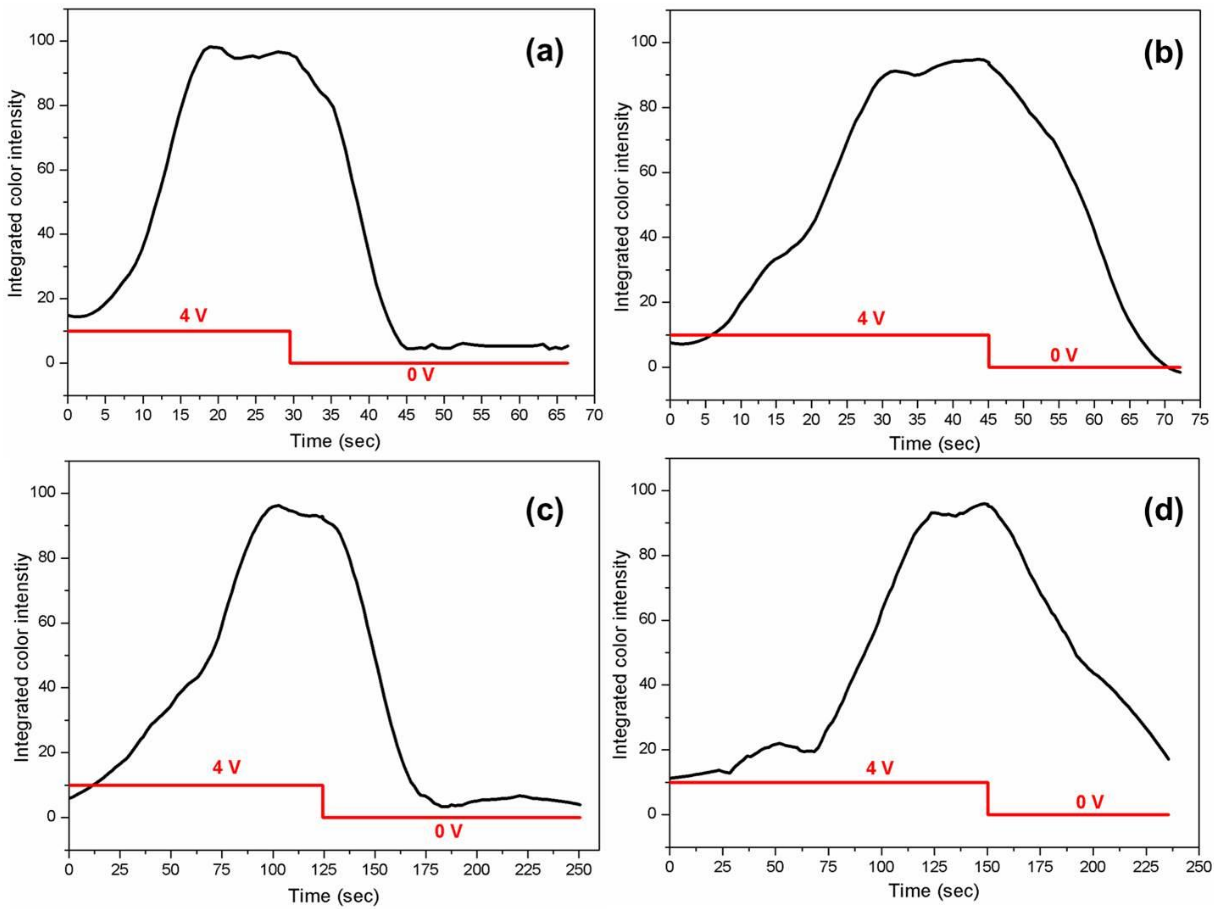

Supplementary Figure 6. Electrochromic switching between colored and colorless state of the DNA-DOAB complex with different DNA lengths in the isotropic liquid phases (45 °C) upon the application of double-potential steps between +4 and 0 V. The electrochromic response time of these DNA materials is 15 s (**a**, 6mer), 30 s (**b**, 14mer), 80 s (**c**, 22mer) and 120 s (**d**, 50mer), indicating DNA length plays an important role in controlling the switching rate. The time-dependent color curves were acquired by analyzing recorded videos with ImageJ software.

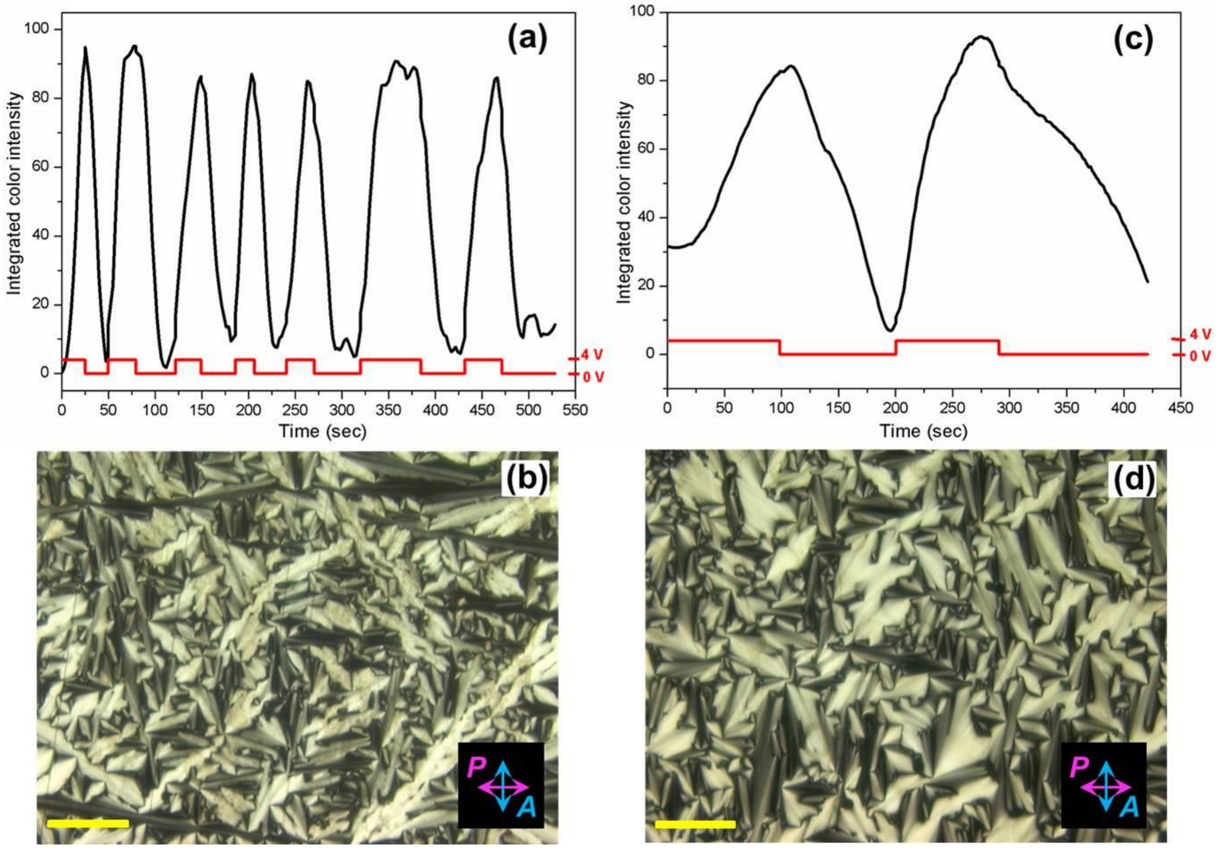

Supplementary Figure 7. Switching response of the electrochromic DNA-DOAB complexes in the liquid phase (45 °C) upon the repeatedly stepping the potential between 4 and 0 V for 30 cycles (**a**, the last seven cycles for 6mer DNA-DOAB; **c**, the last two cycles for 22mer DNA-DOAB). The color switching remained reversible and switch time is comparable with the freshly prepared samples (Supplementary Figure 6). After 30 cycles, the focal-conic birefringent textures were reproduced in the liquid crystal phase (25 °C) (**b**, 6mer DNA-DOAB; **d**, 22mer DNA-DOAB), indicating the redox-active DNA-surfactant materials are stable within this timeframe. Scale bar, 100 µm.

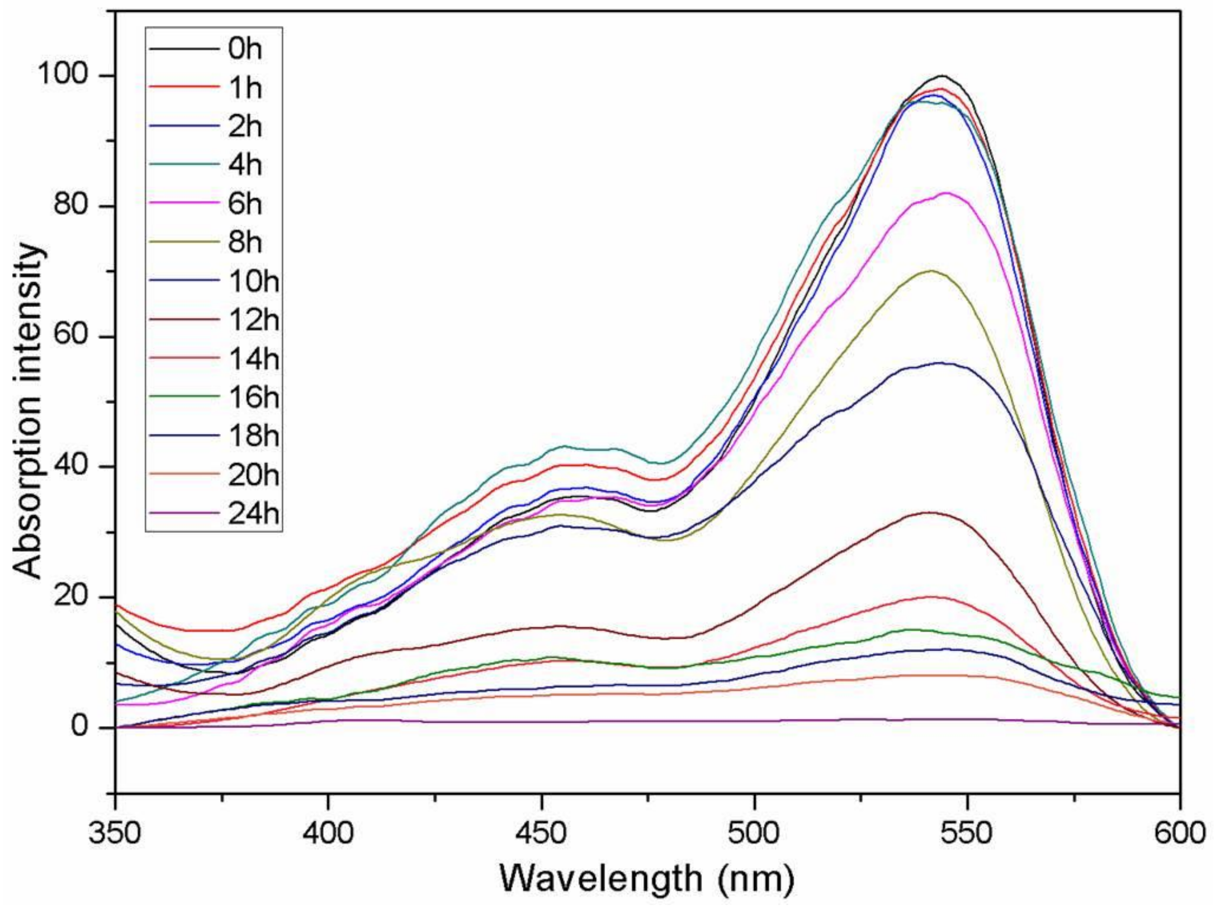

Supplementary Figure 8. Time-dependent optical absorption of the colored DNA-DOAB liquid crystal after bias was removed (corresponding to the treated sample in Figure 2d). The coloration state maintains an intensity within 95% of the original intensity for ~4 hours, indicating its potential as a basis for optical memory devices.

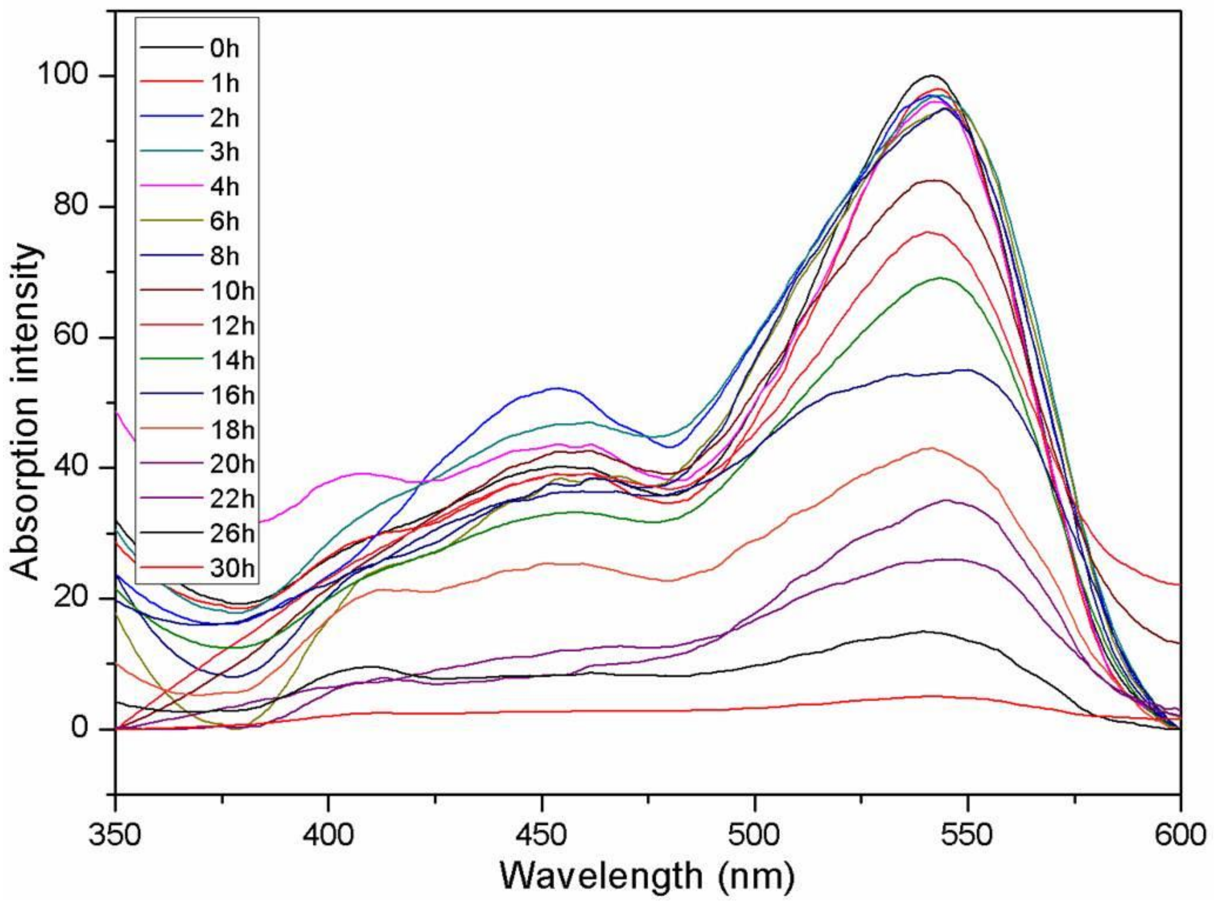

Supplementary Figure 9. Time-dependent optical absorption of the colored DNA-DOAB crystalline phase after bias was removed (corresponding to the treated sample in Figure 2e,2f). The coloration state maintains an intensity within 95% of the original intensity for ~8 hours.

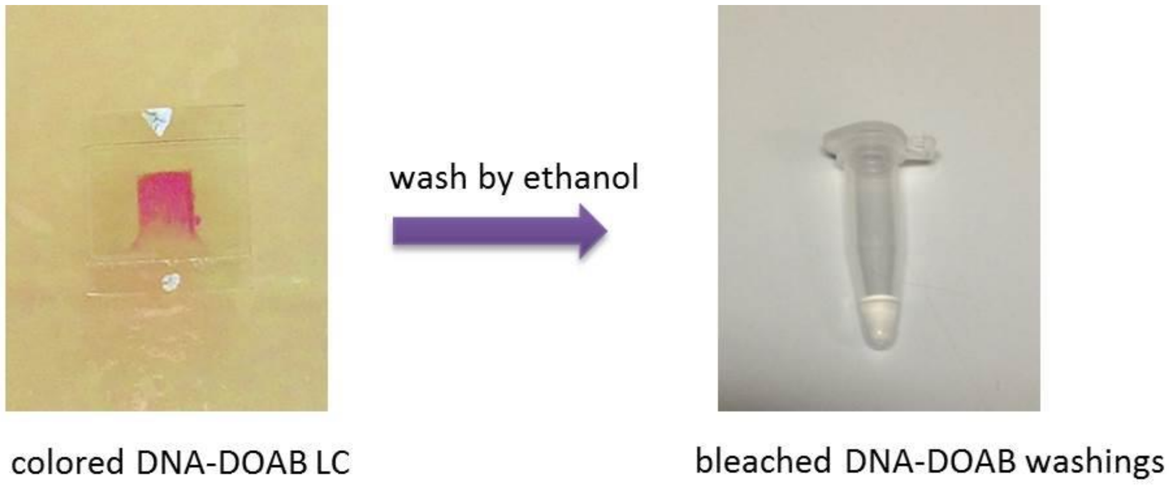

Supplementary Figure 10. Transfer of activated DNA-surfactant complexes from a LC cell to ethanol solution. Bleaching of radical cations processed quickly, probably due to their high mobility in solution.

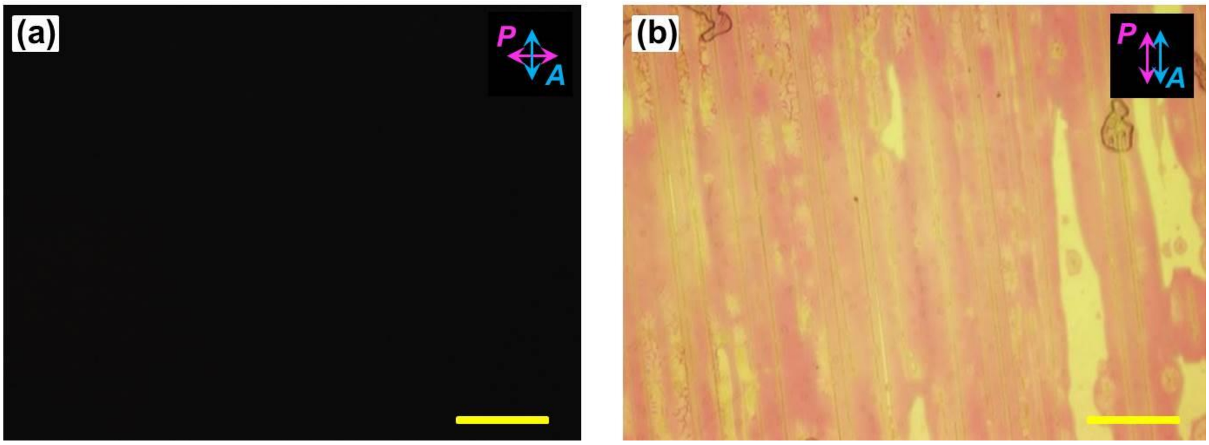

Supplementary Figure 11. Investigation of DNA-DOAB complex in the isotropic liquid phase (45 °C) with an applied voltage of 4 V. No birefringence domains was observed due to the disorder in the material. The DNA radical cations yielded a magenta color.

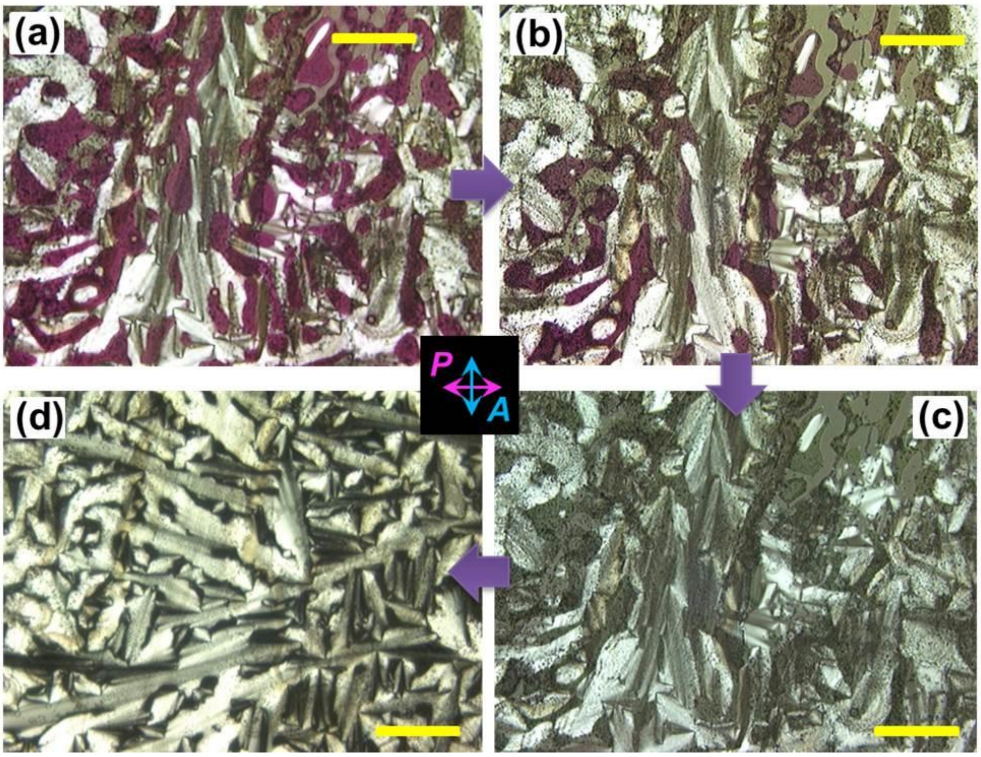

Supplementary Figure 12. Investigation of birefringence textures, orientation, and color-decay of the colored DNA-DOAB complex in the liquid crystalline phase (25 °C) in the absence of applied voltage. The sample was obtained after the many repeated treatment of applying a positive potential in the isotropic state, cooling to the LC state, removing the potential, and again heating to the isotropic state. (**a**) The colored DNA-DOAB smectic layers are orientated parallel to the electrode surface. There is almost no color decay within 4 hours. (**b**) After 15 hours, the color decays a lot. The bleached DNA-DOAB smectic layers keep the parallel alignment. (**c**) After the color decays completely (24 hours), the alignment of the bleached DNA-DOAB lamellar layers remain parallel to electrode surface. (**d**) The completely reproduced birefringent textures with normal perpendicular orientation after annealing treatment of the bleached sample, provides evidence for the stability of the ionic DNA-surfactant complex. Scale bar, 100 µm.

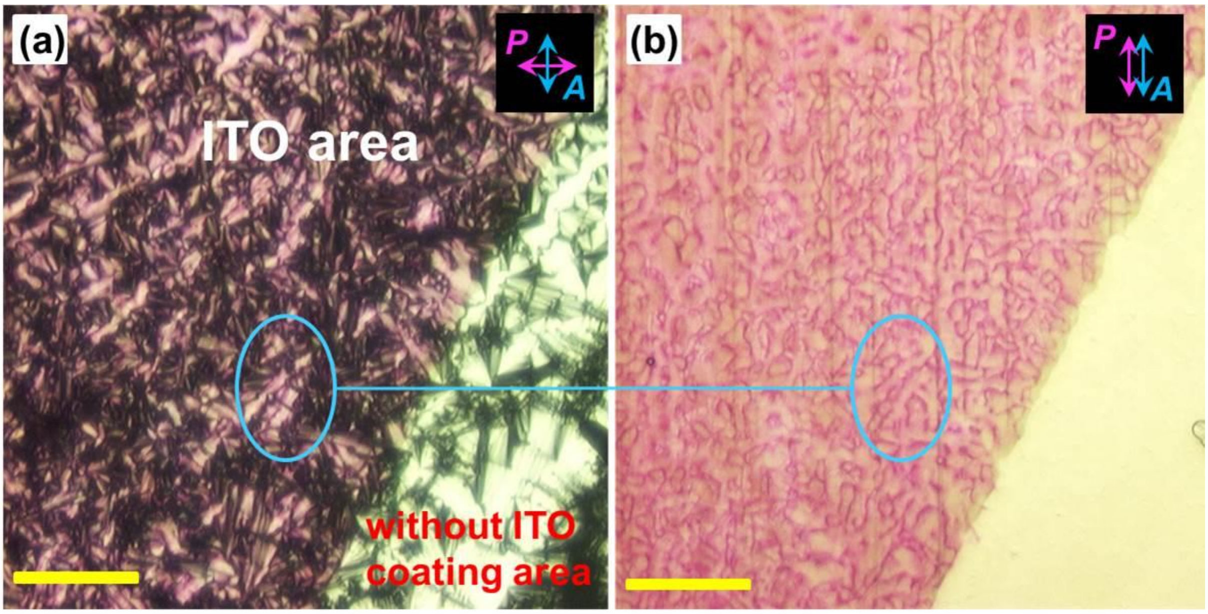

Supplementary Figure 13. Investigation of birefringence textures, orientation, and color domains of the colored DNA-surfactant complex when cooling to the polycrystalline state (-20 °C) in the absence of applied voltage. The POM image of the colored DNA-DOAB textures obtained with crossed polarizer and analyzer (**a**) and the corresponding image (**b**) where the polarizer and analyzer were parallel. Similar to the LC phase, the colored DNA-DOAB smectic layer became parallel to the electrode surface. The bleached DNA-DOAB layers preserved perpendicular orientation. Scale bar, 100 µm.

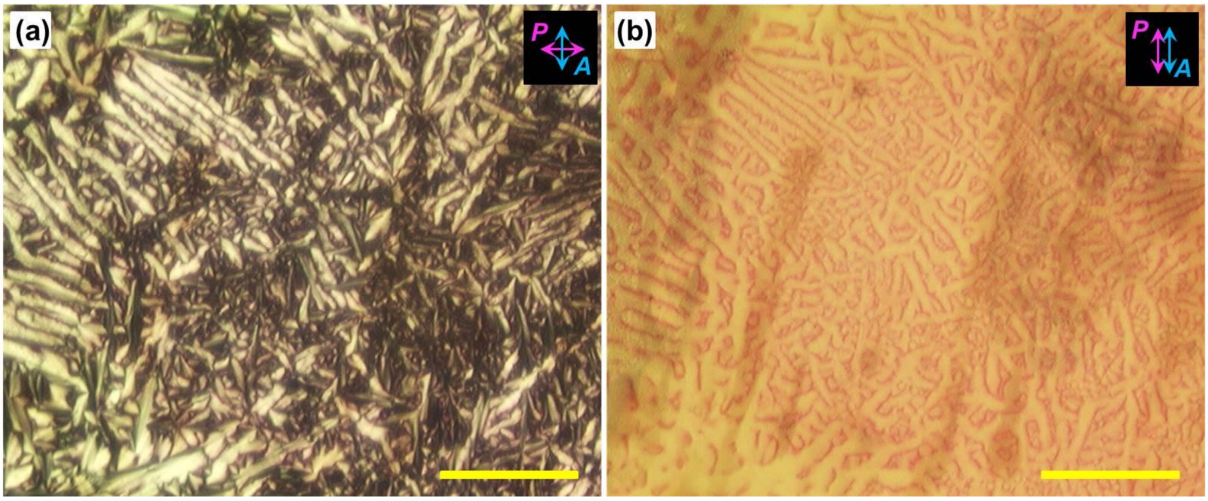

Supplementary Figure 14. Investigation of birefringence textures, orientation, and color domains of the colored DNA-DOAB crystal (-20 °C) after a decay time of 24 hours. The optical image of the DNA-DOAB complex obtained with crossed polarizer and analyzer (**a**) and the corresponding image (**b**) with parallel polarizer and analyzer showed that the orientation of color-decayed DNA-DOAB smectic layers preserved horizontal alignment. Scale bar, 100 μm.

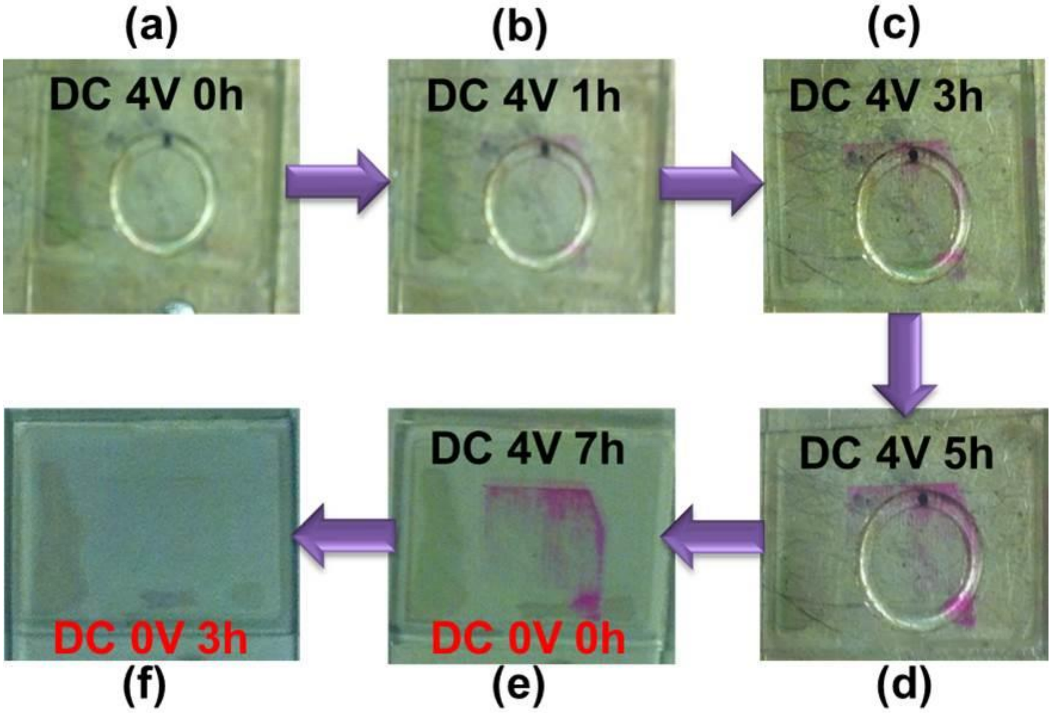

Supplementary Figure 15. Electrochromism (coloration and coloration decay) of the DNA-DOAB complex with voltage (+4 V) applied directly to the LC phase (25 °C). The coloration process proceeds very slowly. The color decay is complete within 3 hours of removing the bias.

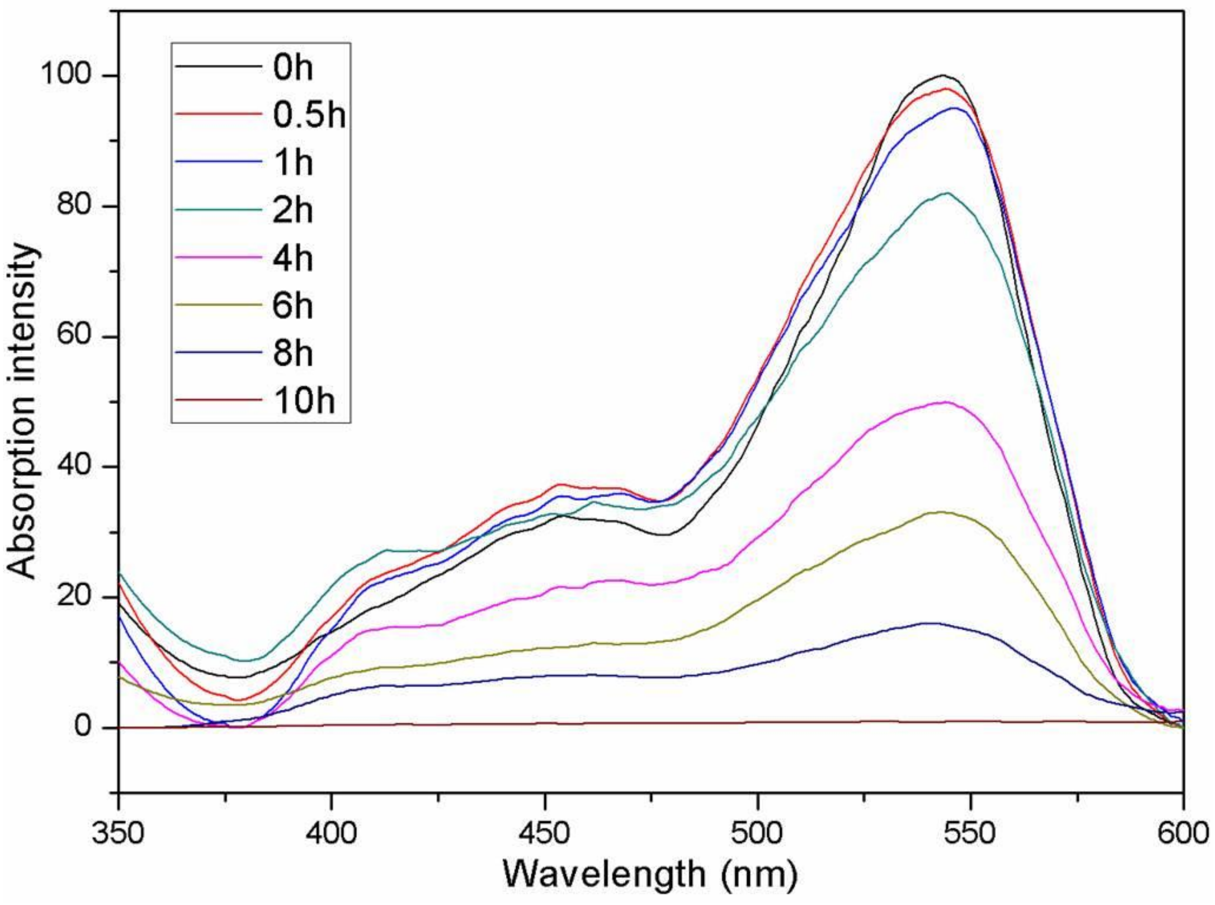

Supplementary Figure 16. Time-dependent optical absorption of the colored DNA-0.5DOAB-0.5DEAB liquid crystal (45 °C) within a given time of removing the bias. The coloration state maintains an intensity within 95% of the original intensity for ~1 hours.

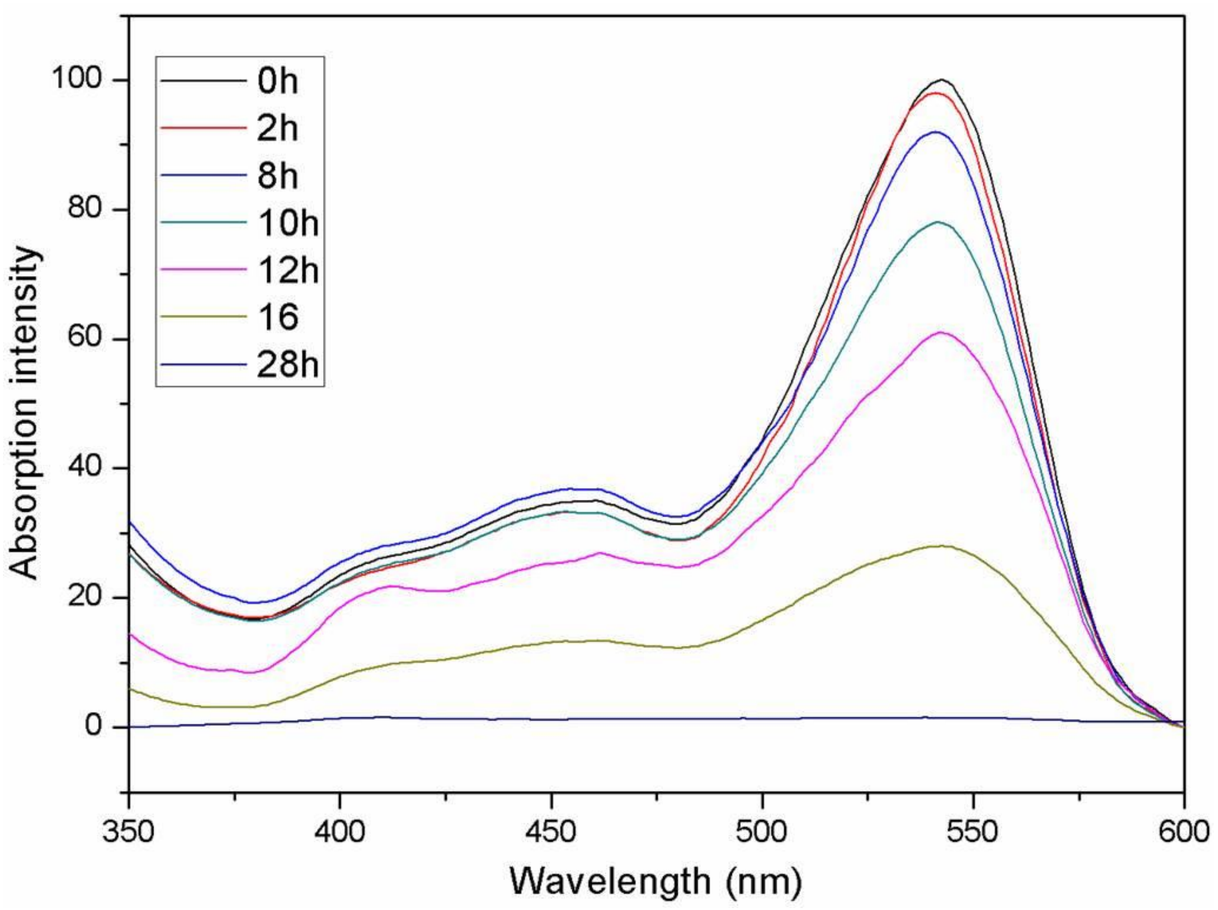

Supplementary Figure 17. Time-dependent optical absorption of the colored DNA-DEAB complex when cooling to the polycrystalline phase (25 °C) in the absence of applied voltage. The coloration state maintains an intensity within 95% of the original intensity for ~ 7.5 hours.

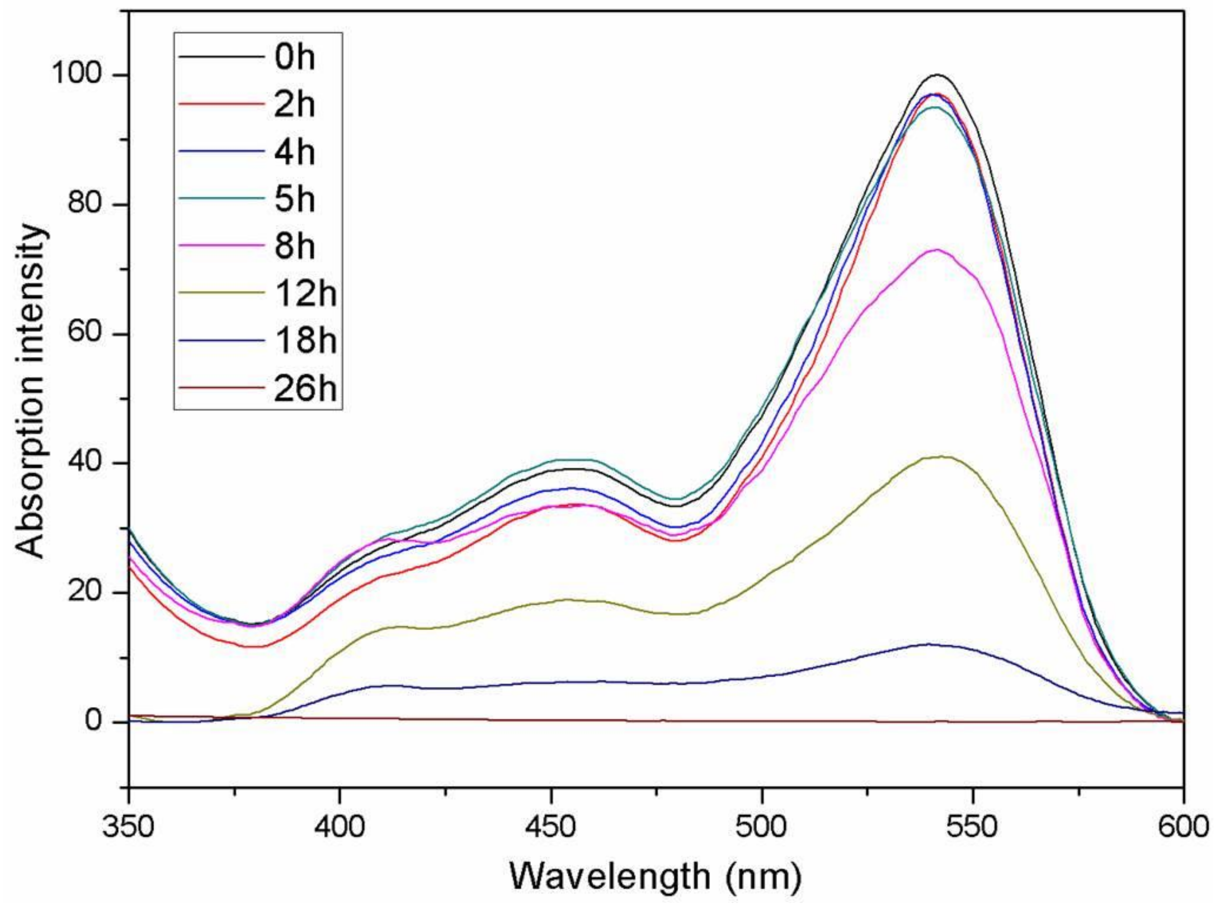

Supplementary Figure 18. Time-dependent optical absorption of the colored DNA-0.5DOAB-0.5DEAB liquid crystal (25 °C) in the absence of applied voltage. The coloration state maintains an intensity within 95% of the original intensity for ~5 hours.

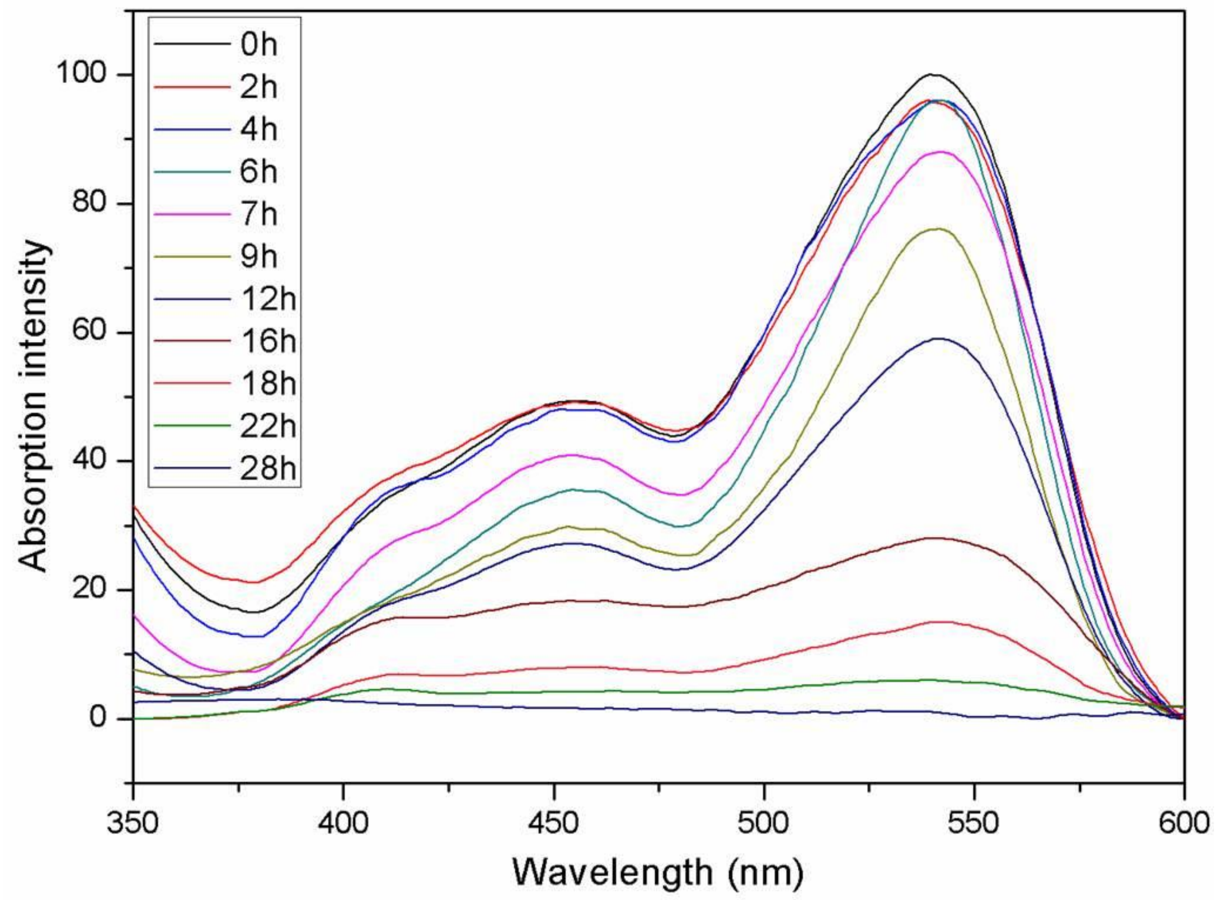

Supplementary Figure 19. Time-dependent optical absorption of the colored DNA-0.5DEAB-0.5DDAB liquid crystal (25 °C) in the absence of applied voltage. The coloration state maintains an intensity within 95% of the original intensity for ~6 hours.

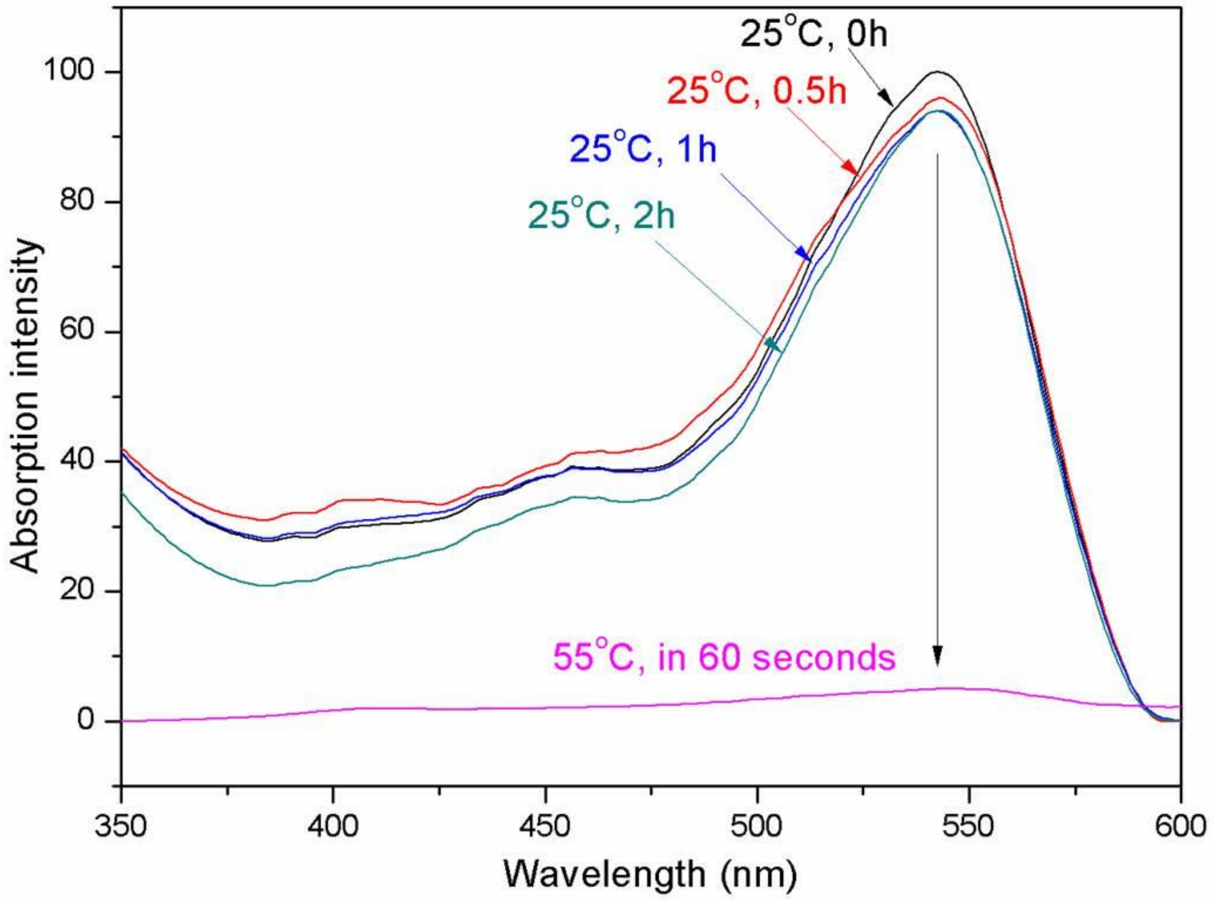

Supplementary Figure 20. Temperature-sensitive optical decay of the colored DNA-DOAB LC sample. At 25 °C, the color-activated sample has no color decay for many hours in the absence of applied voltage. Once the sample was in 55 °C (e.g., after 2 hours of the optical memory device), the color decayed completely in 60 seconds.

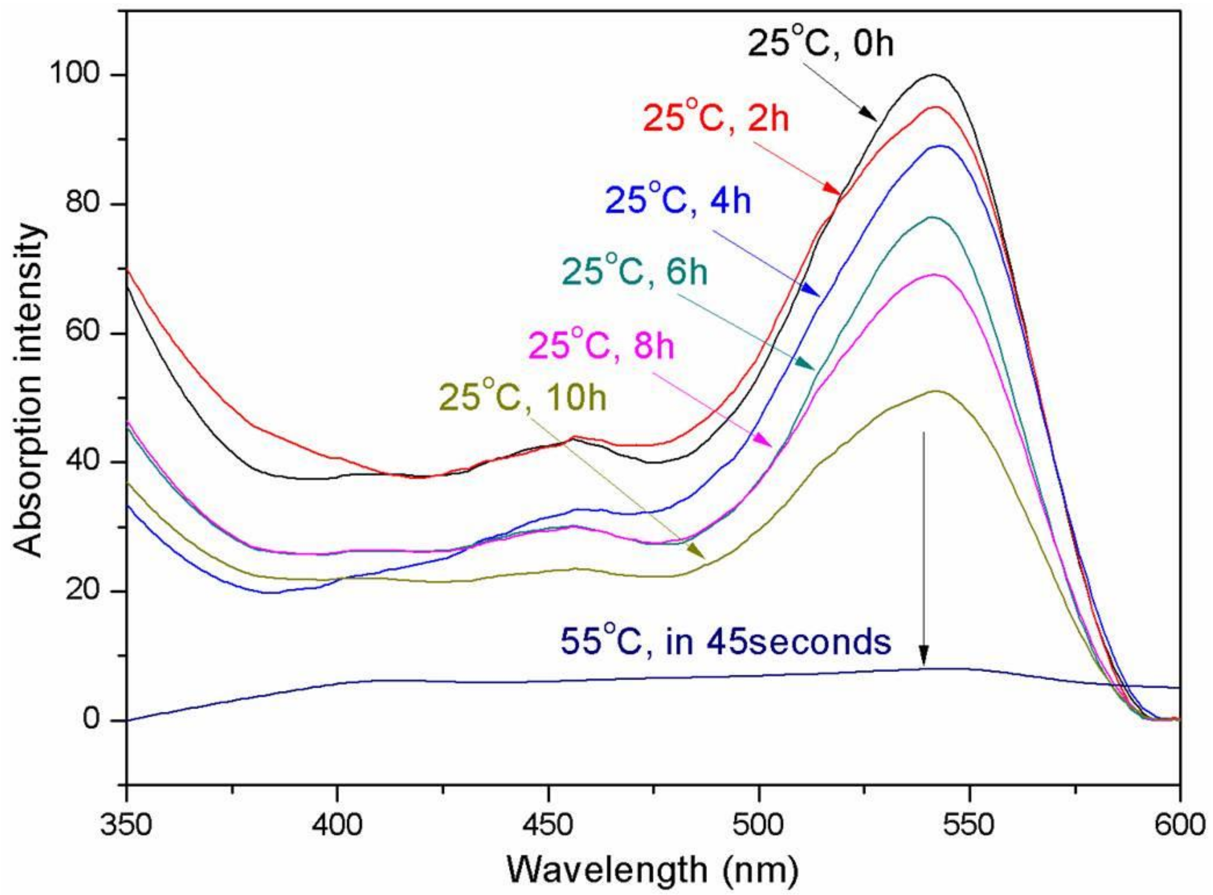

Supplementary Figure 21. Temperature-sensitive optical decay of the colored DNA-DOAB LC sample. At 25 °C, the color-activated sample has very slow color decay in the absence of applied voltage. Once the sample was in 55 °C (e.g., after 10 hours of the optical memory device), the color decayed completely in 45 seconds.

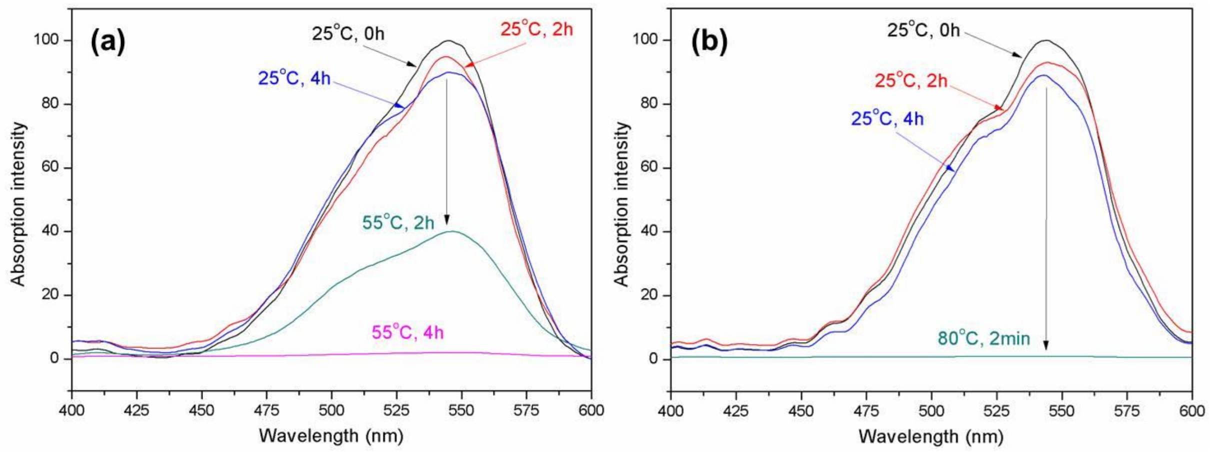

Supplementary Figure 22. Absorption spectra of activated DNA-0.3DOAB-0.7DEAB LC samples at different temperatures. (**a**) When the sample was heated from 25 °C to 55 °C (e.g., after 4 hours in the optical memory device), the color decayed completely in 4 hours. (**b**) When the sample was heated from 25 °C to 80 °C (e.g., after 4 hours in the optical memory device), fast recovery of the colorless state was detected in 120 seconds.

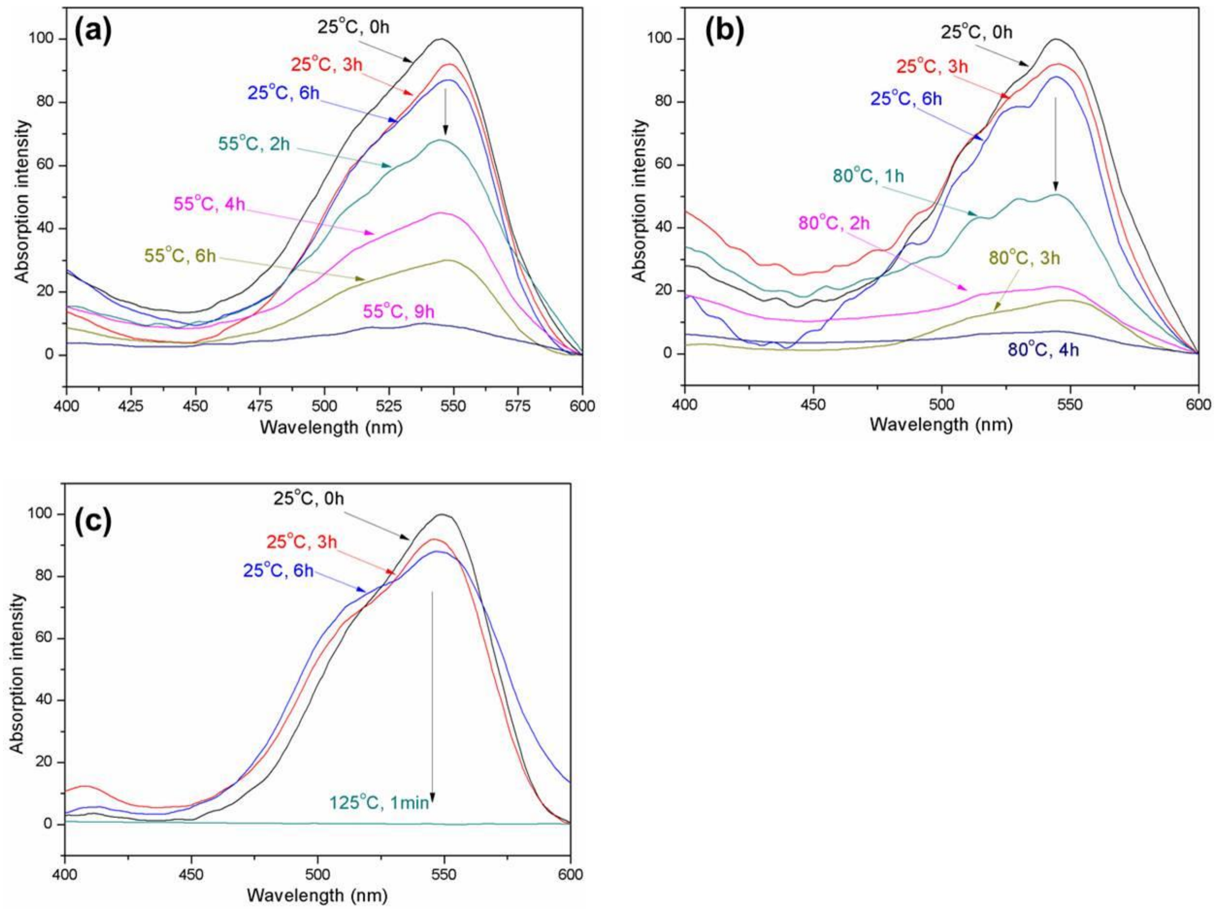

Supplementary Figure 23. Absorption spectra of activated DNA-0.3DEAB-0.7DDAB LC samples at different temperatures. (**a**) When the sample was heated from 25 °C to 55 °C (e.g., after 6 hours in the optical memory device), the color decayed completely in 9 hours. (**b**) When the sample was heated from 25 °C to 80 °C (e.g., after 6 hours in the optical memory device), it showed recovery of the colorless state in 4 hours. (**c**) When the sample was heated from 25 °C to 125 °C, fast recovery of the colorless state was detected in 60 seconds.

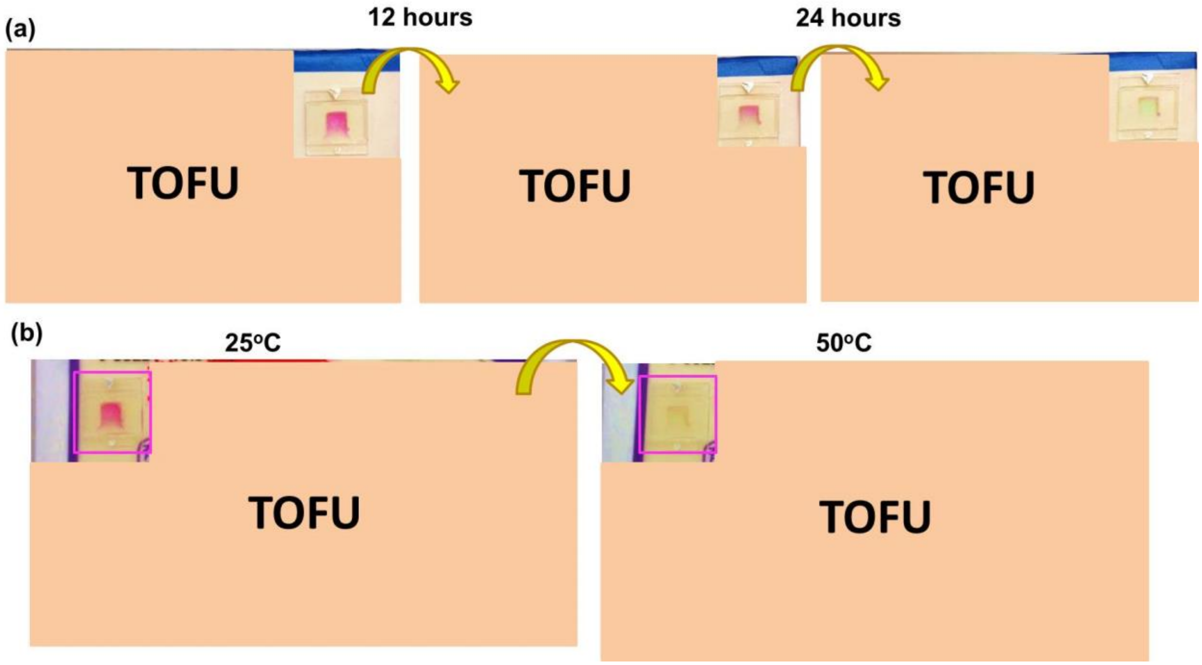

Supplementary Figure 24. Utilization of the color-activated DNA-surfactant LC cell as a smart tag for perishable product inspection. TOFU is a perishable food with an usual lifetime of 24 hours at 25 °C. (**a**) The activated colored DNA-DOAB LC cell was put on the package of TOFU to track time evolution. The decay time of the color of the DNA-DOAB cell was ~24 hours, indicating its functionality. When the DNA-LC tag reached its colorless state, it can be re-activated and used again, demonstrating its recyclability. (**b**) The re-activated DNA-DOAB LC cell was used to track a second sample of TOFU. Once the food was heated to 50 °C, the colored DNA-LC tag became transparent within one minute. Thus, the proper function of the DNA-LC smart tag was proven successfully on a real-world good.

Supplementary References

1. Liu, K. *et al.* Solvent-free Liquid Crystals and Liquids from DNA. *Chem. Eur. J.* **21,** 4898-4903 (2015).

2. Rokhlenko, Y., Cadet, J., Geacintov, N. E. & Shafirovich, V. Mechanistic aspects of hydration of guanine radical cations in DNA, *J. Am. Chem. Soc.* **136,** 5956-5962 (2014).

3. Candeias, L. P. & Steenken, S. Structure and acid-base properties of one-electron-oxidized deoxyguanosine, guanosine, and 1-methylguanosine, *J. Am. Chem. Soc.* **111,** 1094-1099 (1989).
